# *Aspergillus niger* Secretes Citrate to Increase Iron Bioavailability

**DOI:** 10.3389/fmicb.2017.01424

**Published:** 2017-08-02

**Authors:** Dorett I. Odoni, Merlijn P. van Gaal, Tom Schonewille, Juan A. Tamayo-Ramos, Vitor A. P. Martins dos Santos, Maria Suarez-Diez, Peter J. Schaap

**Affiliations:** ^1^Laboratory of System and Synthetic Biology, Wageningen University & Research Wageningen, Netherlands; ^2^Laboratory of Microbiology, Wageningen University & Research Wageningen, Netherlands; ^3^LifeGlimmer GmBH Berlin, Germany

**Keywords:** *Aspergillus niger*, citrate secretion, iron homeostasis, siderophores, metabolic overflow

## Abstract

*Aspergillus niger* has an innate ability to secrete various organic acids, including citrate. The conditions required for *A. niger* citrate overproduction are well described, but the physiological reasons underlying extracellular citrate accumulation are not yet fully understood. One of the less understood culture conditions is the requirement of growth-limiting iron concentrations. While this has been attributed to iron-dependent citrate metabolizing enzymes, this straightforward relationship does not always hold true. Here, we show that an increase in citrate secretion under iron limited conditions is a physiological response consistent with a role of citrate as *A. niger* iron siderophore. We found that *A. niger* citrate secretion increases with decreasing amounts of iron added to the culture medium and, in contrast to previous findings, this response is independent of the nitrogen source. Differential transcriptomics analyses of the two *A. niger* mutants NW305 (gluconate non-producer) and NW186 (gluconate and oxalate non-producer) revealed up-regulation of the citrate biosynthesis gene *citA* under iron limited conditions compared to iron replete conditions. In addition, we show that *A. niger* can utilize Fe(III) citrate as iron source. Finally, we discuss our findings in the general context of the pH-dependency of *A. niger* organic acid production, offering an explanation, besides competition, for why *A. niger* organic acid production is a sequential process influenced by the external pH of the culture medium.

## Introduction

The filamentous fungus *Aspergillus niger* has an innate ability to secrete organic acids in high quantities, and is essential as a biotechnological citrate producer (Schuster et al., 2002). On glucose as carbon source, wild type *A. niger* secretes gluconate and oxalate as well as citrate. To inhibit by-production of gluconate and oxalate, *A. niger* citrate production requires pH ≤ 2.5 and the absence of manganese (Mn^2+^) ions, thereby enforcing overproduction of citrate instead (Currie, 1917; Kubicek and Röhr, 1977; Ruijter et al., 1999). Further production conditions that have been reported to influence external citrate accumulation are, amongst others, the choice and concentrations of the carbon and nitrogen sources, and the concentrations of trace elements in the culture medium (Karaffa and Kubicek, 2003).

Although suggested to be the basis of industrial *A. niger* citrate production (Neilands, 1981), the physiological reason underlying the particular requirement of suboptimal iron concentrations to prompt increased *A. niger* citrate secretion is not yet fully understood. Iron (Fe) is essential for virtually all biological systems. In aerobic environments, ferrous (Fe(II)) iron is oxidized to ferric (Fe(III)) oxyhydroxide polymers (FeOOH), which are stable and have low solubility in aqueous environments, especially at neutral pH (Guerinot and Yi, 1994). Thus, although omnipresent, iron is often biologically unavailable. Iron excess, on the other hand, can be harmful due to the ability of Fe(II) to catalyze the formation of cell-damaging reactive oxygen species, and microbes have thus developed complex systems for tight control over iron uptake and intracellular storage (Haas, 2014).

In fungi, there are four known systems of iron uptake: (i) low-affinity (ferrous) iron uptake, (ii) heme uptake and degradation, (iii) reductive iron assimilation (RIA), and (iv) siderophore mediated (ferric) iron uptake (which, in some cases, involves RIA) (Haas, 2014). Low affinity ferrous iron uptake is less relevant under iron limited conditions, and the *A. niger* heme uptake system has been recently studied (Franken et al., 2014). Iron siderophores are low molecular weight molecules that are secreted to scavenge (ferric) iron from the environment and make it available to the microbe. This can be either by reduction of the siderophore bound Fe(III) to more soluble Fe(II), which is then imported separately from the siderophore (i.e., RIA), or by uptake of the whole iron-siderophore complex (Neilands, 1995).

Iron uptake and siderophore biosynthesis in *Aspergilli* has been found to be under the control of the transcription factors SreA and HapX, which are interconnected in a negative feedback loop (Hortschansky et al., 2007; Schrettl et al., 2008). Under conditions of iron excess, SreA represses HapX as well as the high-affinity iron uptake system and iron siderophore biosynthesis, thereby avoiding the uptake of toxic amounts of iron (Schrettl et al., 2008). Under iron limited conditions, HapX is derepressed, and, in turn, HapX represses SreA as well as iron-dependent pathways (Hortschansky et al., 2007).

With the exception of some budding and fission yeasts, fungi synthesize their own iron siderophores (Haas, 2014). Coprogen B and ferrichrome were identified as two *A. niger* iron siderophores (Franken et al., 2014). However, oxalate and citrate both have inherent chelating properties (Dutton and Evans, 1996; Gadd, 1999), and citrate is an established iron siderophore in various different plant and prokaryotic systems (Cox et al., 1970; Frost and Rosenberg, 1973; Cox, 1980; Guerinot et al., 1990; Silva et al., 2009). Therefore, we hypothesize that, besides coprogen B and ferrichrome, citrate could serve as additional *A. niger* iron siderophore.

In this study, we aim to further elucidate the link between iron limitation and increased *A. niger* citrate secretion. To this end, we measured citrate per glucose production of *A. niger* grown with varying iron concentrations and nitrogen sources, and established a direct link between citrate secretion and iron availability. We investigated the effect of iron limitation on the *A. niger* transcriptome, and found changes associated to biomass, iron siderophore, and citrate and oxalate biosynthesis genes. Finally, we found that *A. niger* can utilize Fe(III) citrate as iron source. Our results support the hypothesis that citrate acts as *A. niger* iron siderophore, and provide insights on why *A. niger* organic acid production is a pH-dependent process.

## Materials and methods

### Strains, media, and culture conditions

The *A. niger* strains N402 (*cspA1*), NW305 (*cspA, goxC17*, Δ*argB*) (Ruijter et al., 2003), and NW186 (*cspA1, goxC17, prtF28*, Δ*argB, pyrA6*), a Δ*argB* and *pyrA6* derivative of NW185 (Ruijter et al., 1999), were used for this study. For the growth experiments, *A. niger* NW186 was transformed as described (Kusters-van Someren et al., 1991) with the plasmid pGW635 (Goosen et al., 1989), carrying the *pyrA* gene of *A. niger*. This complements the *pyrA6* transformation marker and restores the uridine prototrophy. In addition, we measured citrate production under low iron stress (no iron added to the medium, see Supplementary File 1) in *A. niger* NW129 (*cspA1, goxC17, pyrA1*) (Ruijter et al., 1997).

To obtain spores, *A. niger* was grown (from glycerol stock), for 4 days, on complete medium (CM) agar plates, containing, per 1000 mL: 2 g meat peptone, 1 g yeast extract, 1 g casamino acids, 0.3 g yeast ribonucleic acids, 15 g agar and minimal medium (MM) salts (MM salts, per 1,000 mL: 6 g NaNO_3_, 1.5 g KH_2_PO_4_, 0.5 g KCl, and 0.5 g MgSO_4_ 7H_2_O), added before sterilization, and 1 mL vitamin solution (composition of vitamin solution, per 100 mL: 0.01 g thiamine, 0.10 g riboflavin-5P, 0.01 g p-aminobenzoic acid, 0.10 g nicotinamide, 0.05 g pyridoxine-HCl, 0.01 g pantothenic acid, 0.002 g biotin), 1 mL Vishniac (Vishniac and Santer, 1957) solution, 50 mM (9 g) glucose, and 0.02% arginine, added after sterilization. After 4 days of growth, spores were harvested with 12–13 mL of Saline-Tween solution.

A total of 10^6^ spores/mL were inoculated in 1 L Erlenmeyer flasks containing 200 mL of *A. niger* production medium (PM, per 1,000 mL: 1.2 g NaNO_3_ or 0.93g (NH_4_)_2_SO_4_, 0.5 g KH_2_PO_4_, 0.2 g MgSO_4_·7H_2_O), 100 mM (~20 g/L^−1^) glucose, 40 μL adjusted Vishniac (Vishniac and Santer, 1957) solution (with, per 1000 mL: either 1.0 g (+) or 10 g (++) FeSO_4_·7H_2_O, or 0.94 g (+) or 9.75 g (++) C_6_H_5_FeO_7_·H_2_O (Fe(III) citrate), or no Fe (−)), and 0.02 % arginine (NW305, NW186) supplement. Note that, for a better understanding of the system, yeast extract was not used in any of the experiments. No measures were taken to keep the medium completely free from trace amounts of iron. The conditions referred to as −Fe describe a condition in which no iron was added to the medium, and shows the response of *A. niger* to low iron stress rather than the response of this fungus to complete absence of iron.

Time course experiments were performed in duplicates in a shake flask incubator at 30°C and 200 rpm. Supernatant samples were taken every 24 h. Mycelial dry weight was measured after 96 h.

The yeast strains *Cyberlindnera jadinii* DSM 2361, *Cyberlindnera fabianii* CBS 5640, *Hanseniaspora uvarum* CECT 11105, *Kluyveromyces lactis* CBS 739, *Saccharomyces cerevisiae* NCYC 2826, *Wickerhamomyces anomalus* DSM 6766, and *Wickerhamomyces ciferii* CBS 111 were grown (from glycerol stock) on yeast extract peptone dextrose plates (YPD, per 1,000 mL: 10 g yeast extract, 20 g peptone, 20 g glucose and 15 g agar) and, per 1,000 mL: 0.15 g uracil, 0.5 g leucine, 0.075 g tryptophan and 12.5 g histidine supplements. From these plates, overnight cultures were grown in 10 mL liquid YPD medium with the appropriate supplements (see below). The cultures were spun down, washed with sterile demi water, and resuspended in 10 mL demi water. 100 μL of the resuspension was used to inoculate 100 mL Erlenmeyer flasks (in duplicate) containing 20 mL of medium (per 1,000 mL: 20 g glucose, 5 g (NH_4_)_2_SO_4_, 3 g KH_2_PO_4_, and 0.5 g MgSO_4_·7H_2_O, the same supplements mentioned before, and 40 μL Verduyns (Verduyn et al., 1992) trace metal solution, either with or without FeSO_4_·7H_2_O, or 40 μL Verduyns (Verduyn et al., 1992) trace metal solution without FeSO_4_·7H_2_O, but 0.12 mg/L C_6_H_5_FeO_7_·H_2_O (Fe(III) citrate).

### Metabolite analysis using HPLC

Extracellular metabolite concentrations were determined by high-performance liquid chromatography (HPLC). An ICS5000 HPLC (Thermo Scientific), equipped with an Aminex HPX-87H column (BioRad) at 60°C, and coupled to a refractive index detector (Shodex RI-101, sample frequency 5 Hz) and a Thermo UV/VIS detector (λ = 210 nm), was used. Separations were performed by elution with 0.016 N H_2_SO_4_ at a flow rate of 0.6 mL/min. An organic acid standard, containing oxalic acid, citric acid, malic acid, succinic acid and itaconic acid, and a separate glucose standard, with 2, 5, 10, and 20 mM (both organic acid standard and glucose standard), 100 and 200 mM (only glucose standard) were used to calculate calibration curves for quantification of the extracellular metabolite concentrations. Measurement of known concentrations of a Fe(III) citrate standard matched the areas under the peak for the normal citrate standard. Propionic acid (6 mM) was used as an internal standard.

### Siderophore detection

Total siderophore concentration in the *A. niger* culture medium was quantified using the SideroTec Assay™ (Emergen Bio, Maynooth University, Kildare, Ireland), following the protocol provided. The samples were adjusted to the proper pH range for the assay (pH 6-8) by diluting all samples and standards 40:60 with 1M Tris-HCl, pH 7.0 (40 μL Tris-HCl + 60 μL sample/standard). Samples and standards were measured on a microplate reader (Elx808) at OD650.

### RNA isolation and quality control

RNA extraction was performed using the Maxwell® 16 LEV simplyRNA Tissue kit (Promega). Frozen mycelium (≈100 mg) of the sample was submerged in 400 μL Homogenizing buffer supplemented with 8 μL 1-thioglycerol in a 2 mL Lysing matrix C tube (MP), prefilled with a mix of glass beads. Mycelium samples were disrupted using a FastPrep-24 instrument (MP). After disruption all liquid was transferred to a LEV RNA Cartridge. 200 μL lysis buffer was added and the rest of the extraction was performed by a Maxwell MDx AS3000 machine (Promega) following the protocol. RNA integrity and quantity were assessed with an Experion system (Bio-Rad), and only high quality samples (RIN value ≥ 7) were selected. Total RNA was sent directly to BaseClear (Leiden, The Netherlands) for whole transcriptome shotgun sequencing.

### RNA sequencing and quality check

RNA sequencing (RNA seq) and initial quality check was performed by BaseClear, and was reported as follows: “Single-end sequence reads were generated using the Illumina HiSeq2500 system. FASTQ sequence files were generated using the Illumina Casava pipeline version 1.8.3. Initial quality assessment was based on data passing the Illumina Chastity filtering. Subsequently, reads containing adapters and/or PhiX control signal were removed using an in-house filtering protocol. The second quality assessment was based on the remaining reads using the FASTQC quality control tool version 0.10.0.”

### RNA seq data processing

The RNA seq reads were filtered using SortMeRNA v1.9 (Kopylova et al., 2012) and Trimmomatic v0.32 (Bolger et al., 2014). Read mapping against *A. niger* ATCC 1015 (Andersen et al., 2011; Nordberg et al., 2014), was performed using STAR v2.5.0c (Dobin et al., 2013). Gene coverage calculations were performed using BEDTools v2.17.0 (Quinlan and Hall, 2010), and subsequently normalized for the respective library sizes. Differential expression analysis was performed using the R package edgeR (Robinson et al., 2009). RNA seq normalization and differential expression was performed simultaneously for each comparison. Genes with a count per million (CPM) ≥ 1 in at least two samples were considered to be expressed and kept for further analysis. Trimmed mean of M-values normalization was performed as implemented in the R package edgeR. *P*-values were corrected for multiple testing using the Benjamini-Hochberg procedure. The terms “differentially expressed” and “overexpressed” refer to differences in read counts per CDS, and denote a fold-change ≥ 1.5 (FDR ≤ 0.05). A detailed pipeline of the RNA seq data processing can be found in Supplementary File 2. The aligned.bam files have been submitted to the European Nucleotide Archive (ENA), and can be found under the accession number PRJEB20746.

### Enrichment analyses

The protein products of the expressed genes were annotated using PRIAM (Claudel-Renard et al., 2003), and subsequently assigned to KEGG pathway maps (Kanehisa and Goto, 2000; Kanehisa et al., 2015). Metabolic pathway enrichment analysis was performed using the hypergeometric test implementation (“phyper”) of the R software environment (R Core, 2014).

Protein localization prediction was performed with the Softberry protComp tool (www.softberry.com). Enrichment analysis was performed on differentially expressed genes per organelle as described above.

## Results

### *A. niger* iron-dependent biomass and citrate production

**Figure 1:**
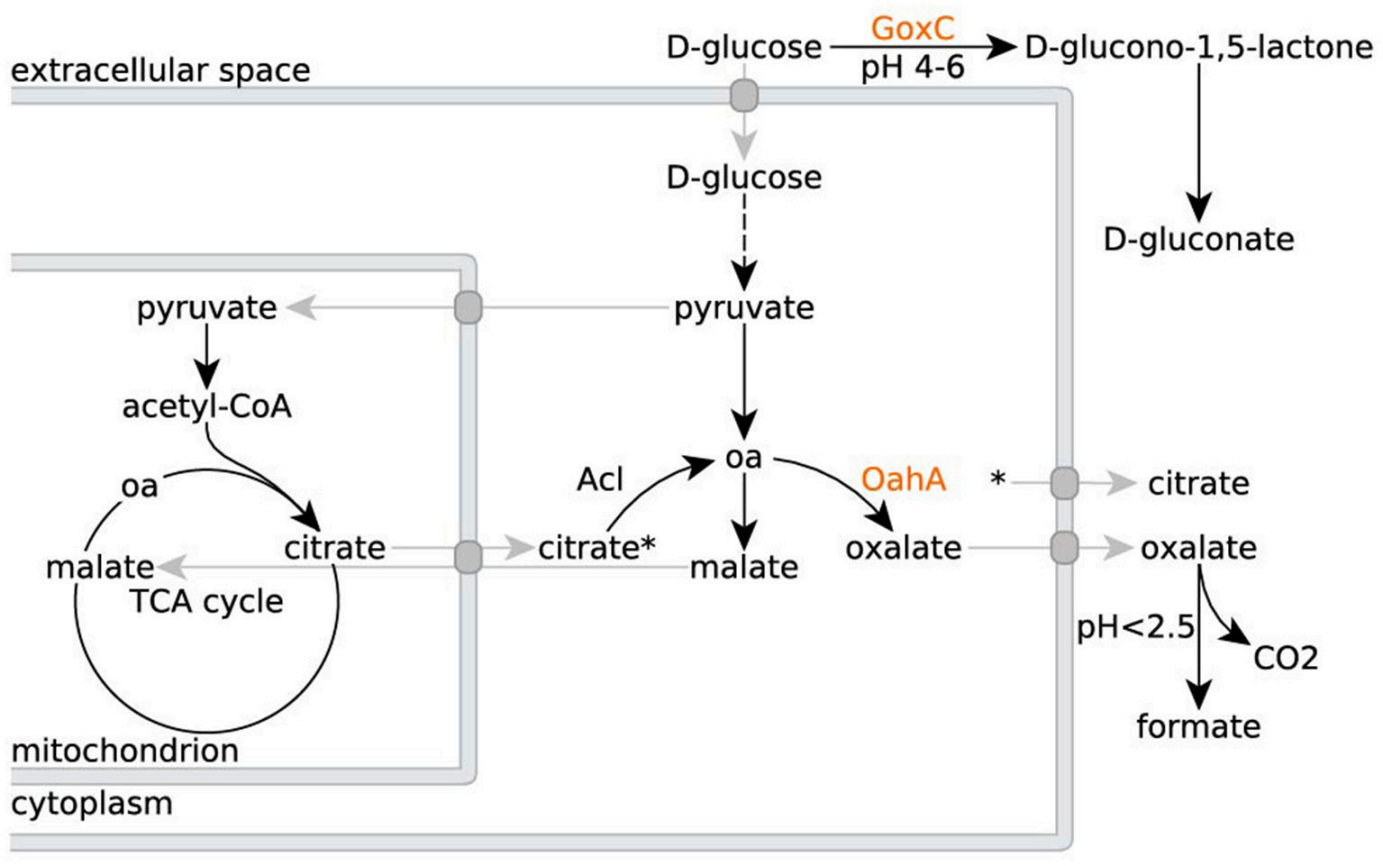
Organic acid production in *A. niger* N402 and its derivatives. Metabolic routes for citrate, oxalate and gluconate production in *A. niger* N402 and its derivatives (oa = oxaloacetate). Mutants defective in GoxC can no longer produce gluconate, whereas mutants defective in OahA can no longer produce oxalate. A hypothetical malate/citrate antiport (Karaffa and Kubicek, 2003), and other transport processes, are depicted in gray. Citrate export is indicated by the asterisk.

When grown in a culture medium with glucose as sole carbon source, wild type *A. niger* strains secrete multiple organic acids (Figure 1). Under moderate acidic conditions (pH 5), mainly oxalic acid is secreted. Additionally, glucose is converted extracellularly to gluconate by a secreted glucose oxidase. Upon further acidification of the medium to a pH below 2.5, citrate becomes the predominantly secreted organic acid (Ruijter et al., 1999). To remove gluconate and oxalate as confounding factors, but without having to exert control over the external pH and thereby influencing iron availability, we worked with *A. niger* strain NW186. This strain bears two mutations, one leading to a frameshift in the gene encoding glucose oxidase (*goxC17*), and a nonsense mutation in the gene encoding oxaloacetate hydrolase (*oahA*), making this strain a gluconate and oxalate non-producer (Ruijter et al., 1999; Han et al., 2007).

Another confounding factor is that iron, zinc, copper, manganese, phosphorus, magnesium, potassium, and nitrogen limitation reportedly all have a stimulating effect on *A. niger* organic acid production (Chesters and Rolinson, 1951; Kubicek and Röhr, 1977). Thus, we grew NW186 with different nitrogen sources, and varying amounts of iron sulfate (Fe(II)SO_4_) added to the medium, but otherwise identical growth conditions (see Materials and Methods). *A. niger* biomass production increases with the amount of iron added to the medium (Table 1) and, in contrast to previous findings (Currie, 1917), this happens irrespective of whether nitrogen is supplied as nitrate (NaNO_3_) or ammonium ((NH_4_)_2_SO_4_). Note that no special measures were taken to keep the cultures completely free from any traces of iron, which will most likely be present. However, the increasing biomass upon addition of more iron to the medium (Table 1) shows that, in our experimental setup, iron is the growth-limiting factor; the observations described are thus a direct effect of the amount of Fe(II)SO_4_ added to the medium.

**Table 1:** Final biomass [g/L] of *A. niger* NW186 and the two control strains N402 and NW305 grown with different nitrogen sources and varying iron concentrations in the medium (Fe source: Fe(II)SO_4_).

**Strain (major organic acid(s) produced)**	**N source: NaNO**_**3**_			**N source: (NH**_**4**_**)**_**2**_**SO**_**4**_		
	**−Fe**	**+Fe**	**++Fe**	**−Fe**	**+Fe**	**++Fe**
N402 (gluconate, oxalate, citrate)	0.39 ± 0.01	0.66 ± 1*e*−3	1.52 ± 3*e*−3	0.51 ± 1*e*−3	1.48 ± 3*e*−3	2.34 ± 0.01
NW305 (oxalate, citrate)	0.97 ± 0.02	1.08 ± 0.04	1.73 ± 0.02	0.91 ± 0.01	1.97 ± 0.02	3.45 ± 1*e*−3
NW186 (citrate)	1.09 ± 1*e*−3	1.58 ± 0.01	1.84 ± 1*e*−3	0.89 ± 0.01	2.17 ± 0.03	3.39 ± 0.08

**Figure 2:**
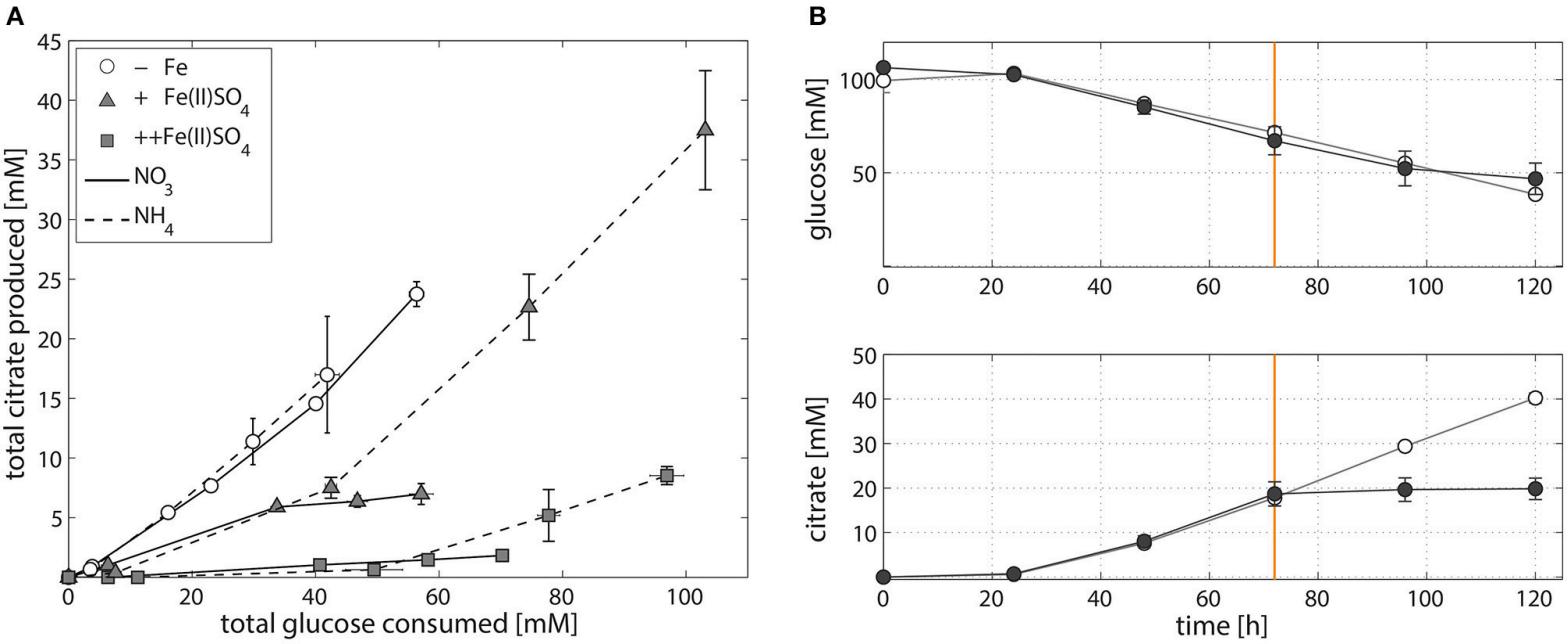
Iron-dependent citrate production of *A. niger* NW186. **(A)** Total citrate production per glucose consumption of *A. niger* NW186, grown without addition of iron (empty circles), or varying amounts of Fe(II)SO_4_ (triangles = iron limited, squares = iron excess), and either NaNO_3_ (solid line) or (NH_4_)_2_SO_4_ (dashed line) as nitrogen source. **(B)** Total glucose and citrate concentration of *A. niger* NW186 grown with (filled symbols) or without (empty symbols) Fe(II)SO_4_ added to the medium at *t* = 72 h (orange line), and NaNO_3_ as nitrogen source. Measurement points were taken once every 24 h and show the average of two biological replicates. The error bars indicate the estimation of standard deviation divided by the number of replicates (N) rather than N-1.

In contrast to the increase in *A. niger* biomass production (Table 1), and again irrespective of whether nitrogen is supplied as NaNO_3_ or (NH_4_)_2_SO_4_, increasing the amount of Fe(II)SO_4_ decreases citrate per glucose production in NW186 (Figure 2A). In addition, we found that NW186 pre-grown without iron stopped secreting citrate after Fe(II)SO_4_ was added to the culture medium (Figure 2B). Final biomass reached 0.93 ± 0.02 g/L and 0.71 ± 1e-3 g/L in the cultures grown with and without Fe(II)SO_4_ added to the medium, respectively.

**Figure 3:**
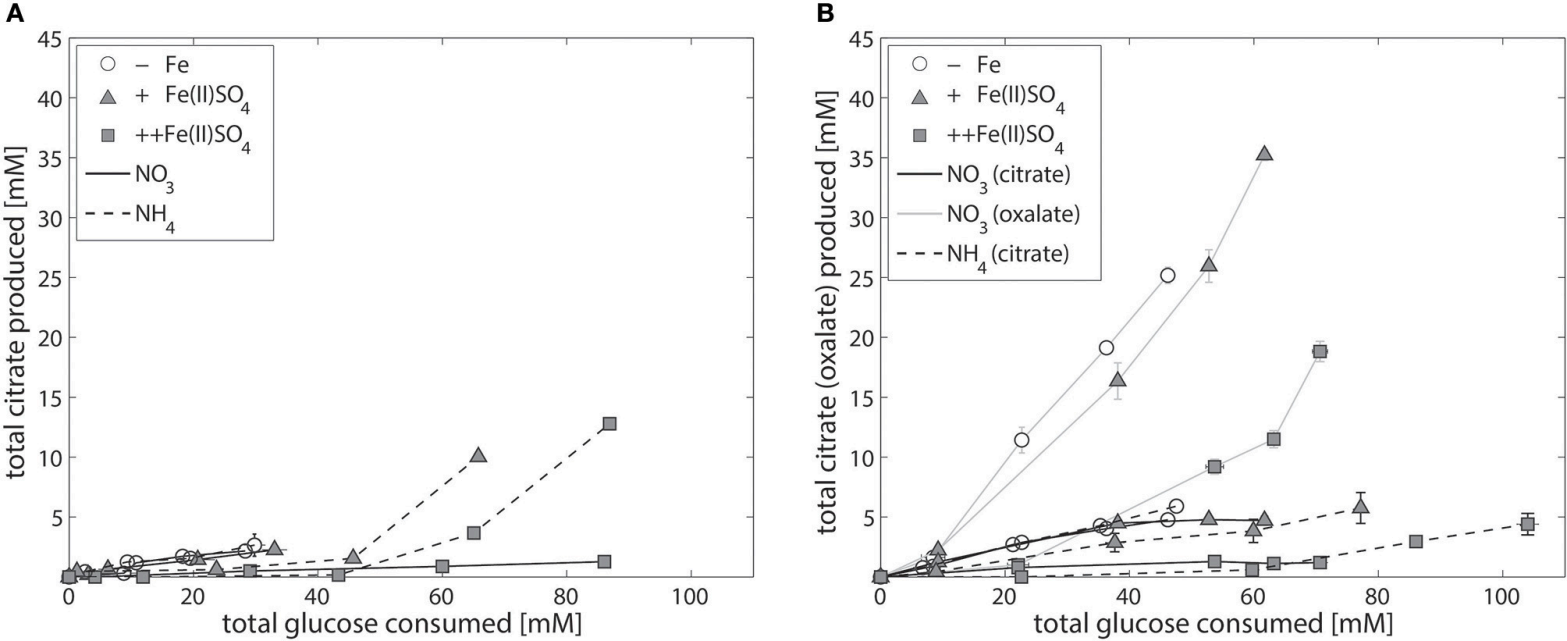
Iron-dependent citrate production of the control strains *A. niger* N402 and NW305. Total citrate production per glucose consumption of the control strains *A. niger* N402 **(A)** and *A. niger* NW305 **(B)**, grown without addition of iron (empty symbols), or varying amounts of Fe(II)SO_4_ (triangles = iron limited, squares = iron excess), and either NaNO_3_ (solid line) or (NH_4_)_2_SO_4_ (dashed line) as nitrogen source. Note that iron-dependent total oxalate per glucose production (gray, solid line) was plotted as example for one experiment (NW305, N source = NaNO_3_). Measurement points were taken once every 24 h and show the average of two biological replicates. The error bars indicate the estimation of standard deviation divided by the number of replicates (N) rather than N-1.

Compared to NW186, decreasing the amount of iron added to the medium did not have an as equally pronounced effect on total citrate per glucose production in the two control strains N402 and NW305 (Figure 3). The direct relationship between iron limitation and citrate secretion is expected to be less straightforward in *A. niger* strains that are able to produce multiple organic acids in major quantities, and the shared relationship of iron on organic acid production is suggested by the secretion pattern of oxalate in NW305, where decreasing iron availability increases both oxalate and citrate per glucose production (Figure 3B).

### *A. niger* iron-dependent gene expression of biomass, iron siderophore, and citrate biosynthesis genes

**Figure 4:**
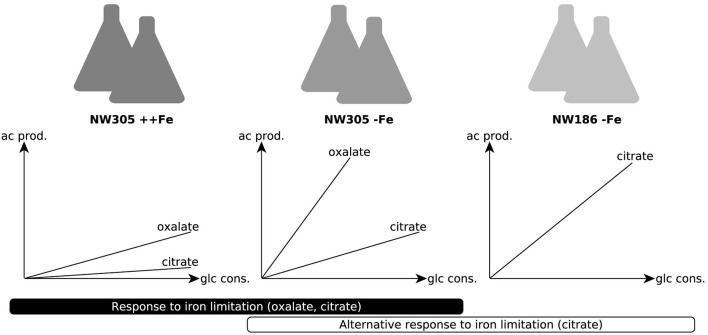
Conditions chosen for differential expression analyses. Experimental setup for RNA seq analyses. The control strain NW305 grown without iron added to the medium was used as reference, and compared to both NW305 grown with excess iron and NW186 grown without iron. The graphs depict the organic acid production profile of the 3 growth conditions. For the *A. niger* response to iron limitation, we compared the transcriptomic landscape of NW305 grown under conditions of iron excess, or without iron added to the medium. The transcriptional response of NW305 grown with or without iron was compared to NW186 grown without iron added to the medium.

The shared relationship of iron on both citrate and oxalate secretion in NW305 (Figure 3B), especially in comparison to the direct relationship of iron on citrate secretion in NW186 (Figure 2A) suggests that increased citrate secretion in NW186 is a result of impaired oxalate secretion due to the OahA mutation. This suggests a lesser role of citrate in wild-type strains of *A. niger*, especially before medium acidification to pH ≤ 2.5, where citrate starts to take over as primary organic acid secreted even in wild type strains (Currie, 1917). To gain further insights into the adaptations to iron limitation, especially with regard to the organic acid secreted, of the oxalate-impaired (hence citrate producing) NW186 mutant, we compared the transcriptional response of NW186 grown without iron added to the medium (NW186 −Fe) to the control strain NW305 grown without iron (NW305 −Fe) or excess iron (NW305 ++Fe) added to the medium (Figure 4).

The RNA seq reads obtained from the different conditions were mapped against the annotated *A. niger* ATCC 1015 genome (Andersen et al., 2011). Of the 11910 ATCC 1015 reference genes, reads were mapped (with a count per million (CPM) ≥ 1) to 9239, 8687 and 8815 genes in NW305 ++Fe, NW305 −Fe and NW186 −Fe, respectively (Table 2). Of the genes expressed, 3332 were differentially expressed (FDR ≤ 0.05) between the control NW305 ++Fe vs. NW305 −Fe, and 1252 were differentially expressed (FDR ≤ 0.05) between NW186 −Fe vs. NW305 −Fe (Table 2, Supplementary File 3).

**Table 2:** RNA seq mapping and differential expression analyses.

**Strain and culture condition**	**NW305 ++Fe**	**NW305 −Fe**	**NW186 −Fe**
# Reads after QC filtering (see Supplementary File 2)	51,965,960 (1)	45,240,789 (1)	43,227,405 (1)
	29,101,217 (2)	52,325,240 (2)	57,754,970 (2)
Uniquely mapped reads (against ATCC1015 CDS)	57.96% (1)	71.49% (1)	68.82% (1)
	61.22% (2)	73.13% (2)	72.70% (2)
# Genes expressed (CPM ≥ 1)	9239	8687	8815
# Genes differentially expressed, log_2_FC threshold ≥ 0.58 (FDR ≤ 0.05)	3332		
		1252	
# EC covered (mapped to KEGG pathways)	465		
		464	
# EC differentially expressed, log_2_FC threshold ≥ 0.58 (FDR ≤ 0.05)	196		
		83	

Conforming the results presented in Table 1, metabolic pathway enrichment analysis (Supplementary File 4) showed that addition of iron to the *A. niger* culture medium has the strongest effect on biosynthesis pathways leading to biomass formation, i.e., starch and sucrose metabolism, and biosynthesis of various amino acids is up-regulated in iron replete vs. iron deplete conditions (i.e., in NW305 ++Fe compared to NW305 −Fe). In addition, fatty acid biosynthesis showed enrichment of differentially expressed genes between NW305 ++Fe and NW305 −Fe. In comparison, the biggest difference between NW186 −Fe and NW305 −Fe was related to lipoic and steroid biosynthesis, and drug and xenobiotics metabolism pathways.

**Figure 5:**
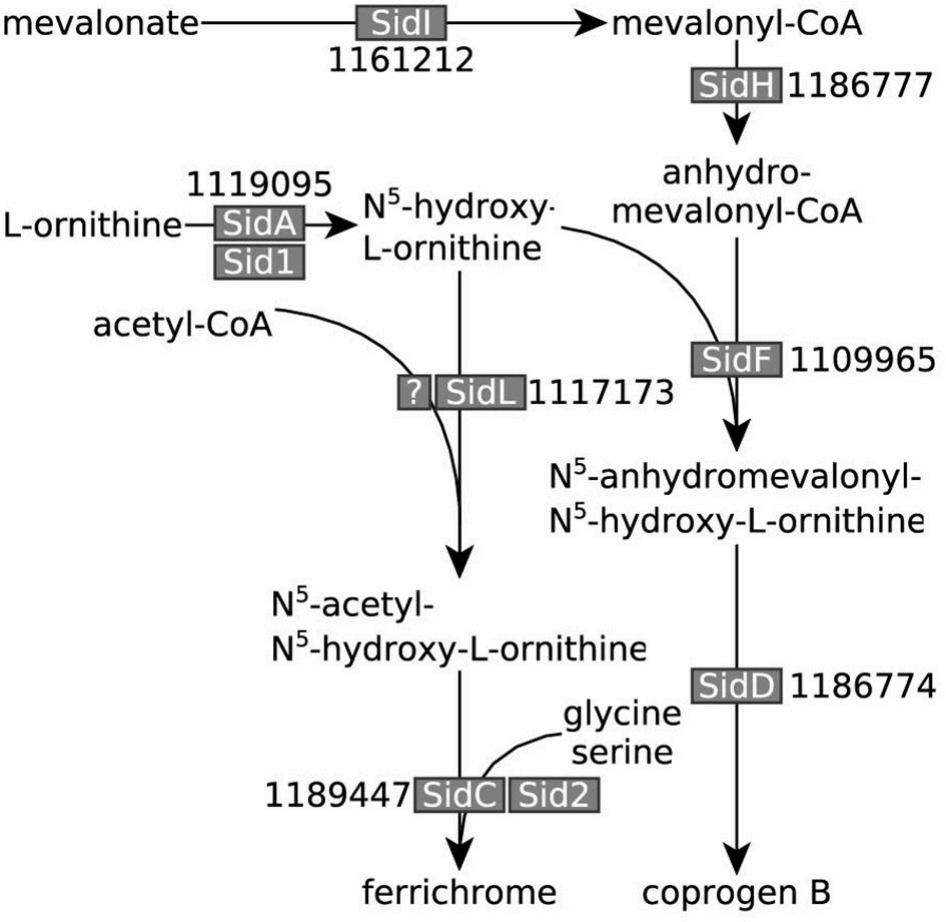
*A. niger* iron siderophore biosynthesis pathway. Iron siderophore biosynthesis genes identified in *A. niger* (adapted from Haas, 2012 and Franken et al., 2014). *A. niger* ATCC 1015 transcript identifiers are denoted next to the names of the enzymes given in other fungi. All genes, except the gene encoding the elusive *A. niger* SidL homolog, were overexpressed under iron limited conditions.

We analyzed expression levels of *A. niger* genes reported to be involved in iron homeostasis and siderophore biosynthesis (Haas, 2012; Franken et al., 2014). In agreement with (Haas, 2003; Franken et al., 2014), *A. niger* iron siderophore biosynthesis shows a clear response to iron limitation at transcriptional level (Figure 5, Supplementary File 5). The difference in iron siderophore biosynthesis is also reflected in the total amount of iron chelating compounds in the *A. niger* supernatant (Table S1 in Supplementary file 6). As can be seen in Table S1, *A. niger* secretes more iron chelating compounds under iron limited conditions, and the concentration of these compounds increases over time.

Note that most of the proteins listed in Supplementary File 5 are based on best bi-directional Blast hits with the *A. niger* CBS 513.88 proteins identified by Franken et al. (2014). We included 1181156 (ATCC 1015 transId 1181432) in the list as possible transcriptional factor related to iron homeostasis and/or organic acid production due to its high expression level and pattern of transcription. 1181156 has a similar basic helix-loop-helix structure as the known *A. fumigatus* transcription factor SrbA, which was found to be essential for adaptation of this fungus to low iron stress (Blatzer et al., 2011). In addition, we included all ATCC 1015 proteins with putative metalloreductase/ferric (chelate) reductase activity in the list (Supplementary File 5), and indicated which enzymes use iron as a co-factor (Supplementary File 3). In the ATCC 1015 *in silico* proteome, there are 163 enzymes that interact with iron as ligand according to the BRENDA database (Schomburg et al., 2004) of which 58 were differentially expressed (FDR ≤ 0.05) between NW305 ++Fe vs. NW305 −Fe, although there was no clear pattern of up- or down-regulation in response to the amount of iron added to the medium (i.e., 23 enzymes were up-regulated in NW305 ++Fe, and 32 were down-regulated; see Supplementary File 3).

Similar as for iron siderophore biosynthesis genes (Figure 5, Supplementary File 5), we found that both citrate synthase (*citA*, EC 2.3.3.1) and *oahA* (EC 3.7.1.1) were transcriptionally up-regulated in response to low iron stress (Table 3). In addition, ATP-citrate lyase (EC 2.3.3.8) and, to a lesser extent, (NAD^+^) isocitrate dehydrogenase were transcriptionally up-regulated in response to low iron stress (Table 3). In contrast, expression of citrate metabolizing enzymes aconitase (EC 4.2.1.3) and (NADP^+^) isocitrate dehydrogenase (NADP-IDH, EC 1.1.1.42) did not show exclusively iron-dependent transcriptional regulation; most expressed isozymes were up-regulated in the reference NW305 −Fe compared to both NW305 ++Fe and NW186 −Fe (Table 3).

**Table 3:** Differentially expressed enzymes involved in citrate and oxalate metabolism.

**ATCC 1015 transcriptId, proteinId**	**Predicted EC number**	**Enzyme name**	**log_2_FC NW305 ++Fe vs. NW305 −Fe (FDR)**	**log_2_FC NW186 −Fe vs. NW305 −Fe (FDR)**
1141647, 1141371	2.3.3.1	Citrate synthase	−1.222 (0.002)	−0.167 (0.949)
1218960, 1218684; 1148603, 1148327; 1181034, 1180758	4.2.1.3	Aconitase	0.237 (0.770); −0.763 (0.114); 0.398 (0.556)	−0.984 (0.124); −0.984 (0.055); −0.841 (0.079)
1148025, 1147749	1.1.1.41	(NAD^+^) isocitrate dehydrogenase	−0.636 (0.226)	0.164 (0.949)
1175666, 1175390	1.1.1.42	(NADP^+^) isocitrate dehydrogenase	−2.033 (0.000)	−1.135 (0.022)
1111634, 1111358; 1147138, 1146862	2.3.3.8	ATP-citrate lyase	−1.381 (0.000); −1.178 (0.005)	−0.026 (0.994); 0.193 (0.934)
1145545, 1145269	3.7.1.1	Oxaloacetate acetylhydrolase	−1.427 (0.000)	0.446 (0.732)

Organelle specific differential expression enrichment analysis revealed the biggest difference between the 3 conditions was found in the plasma membrane (Table 4). Additionally, we observed enrichment of differentially expressed peroxisomal proteins in response to iron availability in the medium, i.e., in NW305 ++Fe vs. NW305 −Fe, but not in NW186 −Fe vs. NW305 −Fe (Table 4). These differences could be attributed to the finding that many enzymes that participate in fungal iron siderophore biosynthesis are located in peroxisomes (Gründlinger et al., 2013). In addition, peroxisomes participate in metabolism of oxygen metabolites (Schrader and Fahimi, 2006). Under iron excess, low affinity ferrous iron uptake might lead to an excess of Fe(II) in the cell, which can catalyze the Fenton reaction: Fe(II) + H_2_O_2_ → Fe(III) + OH^−^ + ^·^OH. The resulting oxygen radical ^·^OH can have cell-damaging effects (Schrader and Fahimi, 2006). However, if H_2_O_2_ can be decomposed to O_2_ and H_2_O by gluthatione-peroxidase (EC 1.11.1.9) or catalase (EC 1.11.1.6), the formation of ^·^OH can be prevented (Schrader and Fahimi, 2006). Although we could not identify an ATCC 1015 glutathione-peroxidase, there are 9 predicted catalases in the *A. niger* ATCC 1015 reference proteome (Supplementary File 3), of which 4 are predicted to be located in the peroxisome (1201726, not differentially expressed; 1116766, overexpressed in ++Fe vs. −Fe; 1119521, overexpressed in ++Fe vs. −Fe; 1158108, not differentially expressed), 3 are predicted to be cytosolic (1155727, not differentially expressed; 1137750, underexpressed in ++Fe vs. −Fe; 1228383, not differentially expressed) and one each mitochondrial (1181451, overexpressed in ++Fe vs. −Fe) or secreted (1204436, overexpressed in ++Fe vs. −Fe). Another peroxisomal enzyme that had an interesting expression pattern was pyruvate oxidoreductase (EC 1.2.7.1, transcriptId: 1162221, proteinId: 1161945), which was overexpressed in both NW186 −Fe and NW305 ++Fe compared to NW305 −Fe (Supplementary file 3).

**Table 4:** Organelle specific differential expression enrichment analysis.

**Organelle**	**# Genes expressed (CPM ≥ 1)**	**# Genes differentially expressed, log_2_FC threshold ≥ 0.58 (FDR ≤ 0.05)**	***p*-value**
**NW305 ++Fe vs. NW305 −Fe (*N* = 5658, *kx* = 2231)**			
Cytoplasm	1988	788	0.42
Endoplasmic reticulum	363	123	0.99
Golgi	227	78	0.95
Mitochondrion	1794	684	0.92
Peroxisome	146	68	0.05
Plasma membrane	1140	490	3e-3
**NW186 −Fe vs. NW305 −Fe (*N* = 5641, *k* = 852)**			
Cytoplasm	1993	279	0.96
Endoplasmic reticulum	362	46	0.92
Golgi	224	35	0.44
Mitochondrion	1790	246	0.98
Peroxisome	145	26	0.20
Plasma membrane	1127	220	4e-6

### Fe(III) citrate as iron source for *A. niger* and yeast-type fungi

**Figure 6:**
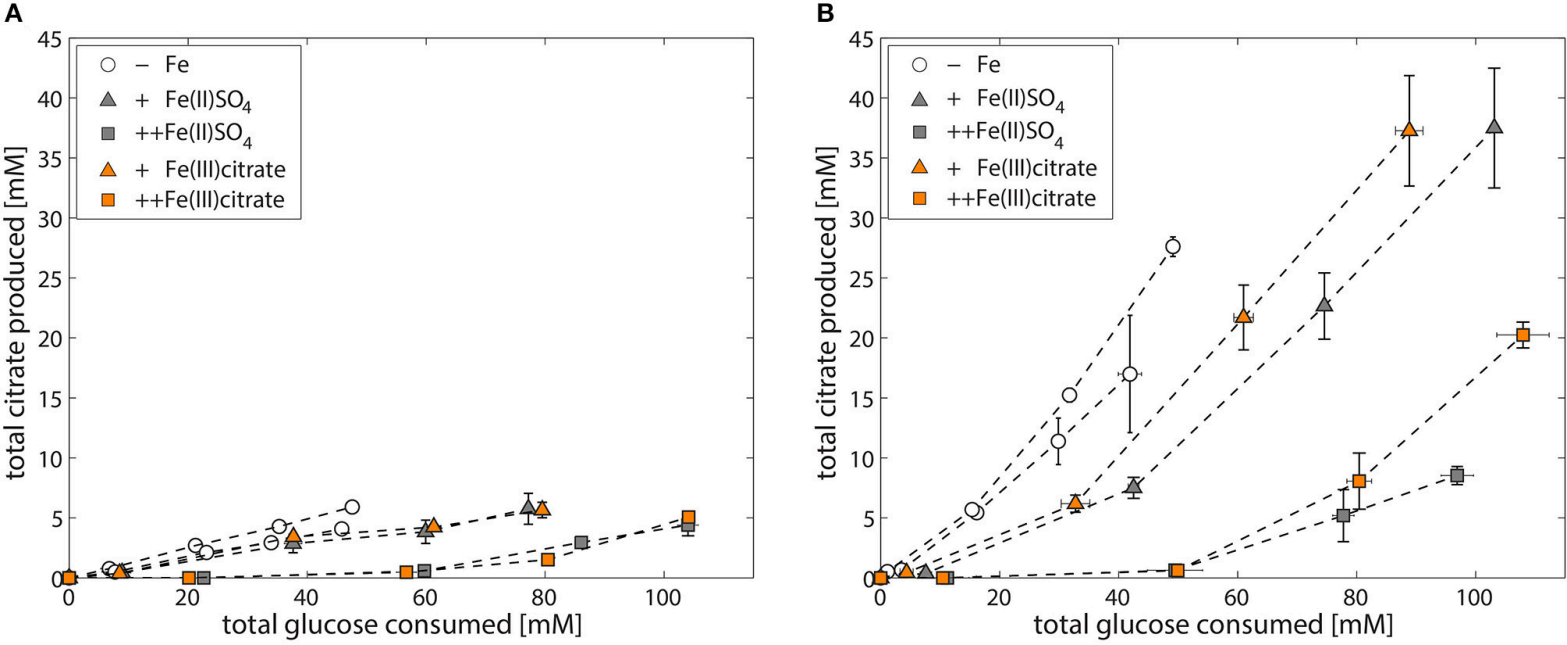
Iron-dependent citrate production of *A. niger* NW305 and NW186 (N source = (NH_4_)_2_SO_4_). Total citrate production per glucose consumption of *A. niger* strains NW305 **(A)** and NW186 **(B)**, grown without addition of iron (empty circles), or varying amounts of iron (triangles = iron limited, squares = iron excess), added either as Fe(II)SO_4_ (gray, filled symbols) or Fe(III) citrate (orange, filled symbols).

Finally, we investigated whether *A. niger* has the means to deal with citrate bound iron as iron source, and found that addition of Fe(III) citrate to the medium alleviates iron limitation and restores the growth phenotype (Table 5). In addition, the amount of iron added as Fe(III) citrate has the same effect on citrate production per glucose consumed as observed with Fe(II)SO_4_ (Figure 6).

**Table 5:** Final biomass [g/L] of *A. niger* NW305 and NW186 grown with Fe(III) citrate as iron source (N source = (NH_4_)_2_SO_4_).

**Strain (major organic acid(s) produced)**	**−Fe**	**+Fe**	**++Fe**
NW305 (oxalate, citrate)	0.78 ± 4e-3	1.86 ± 0.01	3.28 ± 0.01
NW186 (citrate)	0.84 ± 3e-3	1.91 ± 0.03	3.09 ± 0.01

Most budding and fission yeasts, which do not produce own siderophores, are able to utilize iron complexed to xenosiderophores (Haas, 2014). To verify that Fe(III) citrate is a viable iron source for different yeast type fungi, we also tested the ability of *Cyberlindnera jadinii, Cyberlindnera fabianii, Hanseniaspora uvarum, Kluyveromyces lactis, Saccharomyces cerevisiae, Wickerhamomyces anomalus*, and *Wickerhamomyces ciferii* to grow on Fe(III) citrate as iron source. With the exception of *C. jadinii*, which could not grow when Fe(III) citrate was added to the medium, all the strains were able to grow in all the conditions tested (either Fe(II)SO_4_, Fe(III) citrate or no iron added to the medium).

## Discussion

### Citrate as overflow metabolite vs. citrate as biological asset

There are two possible interpretations for the results presented in Figure 2: (i) *A. niger* citrate production is a result of metabolic overflow triggered by carbon excess relative to low iron availability, or (ii) *A. niger* citrate production is a strategy to increase bioavailability of iron. The effect is essentially the same, only in hypothesis (i), citrate is regarded as a “waste product” for the fungus, whereas in hypothesis (ii), citrate is regarded as a biological asset enabling *A. niger* to cope with low iron stress.

To test which of these two hypotheses is more likely, we compared the responses of NW186 and NW305 to varying iron concentrations in the medium. Both of these strains are gluconate non-producers, and NW186 differs from its oxalate producing equivalent NW305 only by dysfunctional OahA (Ruijter et al., 1999; Han et al., 2007). This mutation deprives NW186 of the possibility to produce oxalate *via* the cytosolic route in which oxaloacetate is hydrolysed to oxalate and acetate (Figure 1); the established route of oxalate biosynthesis on glucose as carbon source (Kubicek et al., 1988). As a result, NW186 produces only citrate in major quantities, and decreasing the amount of iron added to the medium is directly reflected in increased citrate per glucose production in this mutant (Figure 2). In NW305, decreasing the amount of iron added to the medium increases both oxalate and citrate per glucose production (Figure 3).

According to hypothesis (i), citrate would thus be regarded as an overflow metabolite alternative to oxalate, implying that carbon flow directed toward oxalate in NW305 stops short at citrate in NW186, and that increased extracellular oxalate secretion by NW305 −Fe is preceded by intracellular citrate accumulation. However, it has been shown that inhibition of *A. niger* aconitase does not affect oxalate production (Kubicek et al., 1988), nor does deletion of ATP-citrate lyase (Acl, Figure 1) (Meijer et al., 2009). In contrast, both of these modifications affect *A. niger* citrate production (Kubicek et al., 1988; Meijer et al., 2009). The contrasting effects of these modifications on citrate and oxalate production suggest that it is unlikely that a substantial amount of oxalate is derived from citrate as precursor, which is also in agreement with (Kubicek et al., 1988). Hypothesis (i) is thus inconsistent with the observed *A. niger* phenotypes (Figures 2, 3).

In the context of the discussion whether inhibition of TCA enzymes downstream of citrate is required for extracellular *A. niger* citrate accumulation, it is noteworthy to mention the aconitase iron-dependency (La Nauze, 1966; Kubicek and Röhr, 1977, 1978; Meixner-Monori et al., 1985; Szczodrak and Ilczuk, 1985). Aconitase catalyzes the conversion of citrate to isocitrate *via* cis-aconitate. Aconitase inhibition due to lack of iron co-factor would explain the observed increase in citrate secretion upon iron limitation. In addition, it has been shown that aconitase, as well as other iron-dependent enzymes, is subject to HapX repression in *A. nidulans* (Hortschansky et al., 2007). However, *A. niger* aconitase, and other TCA cycle enzymes downstream of citrate, have been found to be active during citrate production, even when iron is not added to the medium (La Nauze, 1966; Kubicek and Röhr, 1985; Szczodrak and Ilczuk, 1985; Karaffa and Kubicek, 2003).

**Figure 7:**
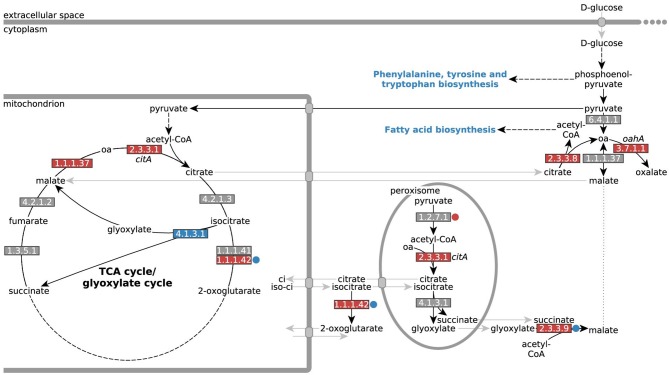
Differential expression of genes encoding enzymes involved in *A. niger* citrate and oxalate metabolism. Differential expression of genes directly involved in A. niger citrate and oxalate metabolism (oa = oxaloacetate). EC numbers are colored based on over- (red) or under-expression (blue) in response to iron limitation, i.e., in the control strain NW305 −Fe vs. NW305 ++Fe. Small circles next to the EC numbers indicate that these reactions were differentially expressed in NW186 −Fe vs. NW305 −Fe. Note that CitA (EC 2.3.3.1) has been found in mitochondria during citrate production, but has a peroxisomal as well as a mitochondrial targeting sequence, and could therefore be multilocated (Jaklitsch et al., 1991).

According to hypothesis (ii) citrate is taking over the biological role of oxalate to increase bioavailability of iron. This hypothesis seems plausible, given that both oxalate and citrate have iron chelating properties (Gadd, 1999). We found that, similarly to iron siderophore biosynthesis pathways (Figure 5) leading to the *A. niger* iron siderophores coprogen B and ferrichrome (Franken et al., 2014), low iron stress is reflected in up-regulation of citrate and oxalate biosynthesis genes *citA* and *oahA*, respectively (Table 3, Figure 7).

The role of CitA during citrate production has led to some controversy. CitA catalyzes the condensation of acetyl-CoA with oxaloacetate to form citrate and coenzyme A, and has been shown to have peak activity during *A. niger* citrate production (Kubicek and Röhr, 1977). However, up to 11-fold overproduction of CitA did not lead to the expected increase in *A. niger* citrate production (Ruijter et al., 2000). As a response to this work, this was later attributed to the activity of CitA within unmodified cells already being well above the activity which would account for the observed rate of *A. niger* citrate production (Ratledge, 2000; Ratledge and Ruijter, 2000). In another instance, the two mitochondrial citrate synthases in *A. niger* H915-1 were even found to be down-regulated during the citrate production phase (Yin et al., 2017).

In agreement with (Ratledge, 2000; Ratledge and Ruijter, 2000; Ruijter et al., 2000; Yin et al., 2017), we found that the difference in extracellular citrate accumulation was remarkably independent of *citA* expression; the gene was not differentially expressed between NW186 −Fe and NW305 −Fe (Table 3, Figure 7). Most remarkably, though, *citA* was overexpressed in both NW186 −Fe and NW305 −Fe compared to NW305 ++Fe, while citrate metabolizing enzymes aconitase (EC 4.2.1.3) and NADP-IDH (EC 1.1.1.42) did not show iron-dependent transcriptional regulation (Table 3, Figure 7). Taken together, these observations suggest that citrate biosynthesis is actively up-regulated in response to iron limitation, but that there is another step of control determining whether citrate is ultimately metabolized (in the case of NW305 −Fe), or secreted (in the case of NW186 −Fe).

Organelle specific differential expression analysis shows the biggest differences in expression levels of plasma membrane proteins between all 3 conditions (Table 4). Therefore, it is likely that extracellular citrate accumulation is ultimately controlled at the transporter level. Transport of citrate was previously hypothesized to be the bottleneck of *A. niger* citrate production (Karaffa and Kubicek, 2003). In our case, we suggest that citrate is only secreted when there is a need to, i.e., in an attempt to increase iron availability under iron limited conditions, and when oxalate is not available for this purpose. Controlling the secretion of citrate irrespective of intracellular citrate biosynthesis, but rather based on the need to increase bioavailability of iron, offers another explanation why overexpression of *citA* does not, *per se*, lead to increased extracellular citrate accumulation (Ruijter et al., 2000). Note that the *A. niger* citrate exporter has, to date, not been identified, but that a list of citrate transporter candidates is provided by Yin et al. (2017).

### Fe(III) citrate as iron source for *A. niger* and yeast-type fungi

The Fe(III) citrate stability constant of 11.85 [expressed as log (Furia, 2006)], although low in comparison to other microbial iron siderophores (Neilands, 1981), suggests that little metal is released from the complex, even at a low pH. Thus, if *A. niger* lacks the means to deal with iron as metal ion complex with citrate, citrate secretion under low iron stress would effectively lead to an even more drastic iron shortage for the fungus. However, addition of Fe(III) citrate to *A. niger* culture medium restores the growth phenotype (Table 5, Figure 6), implying that, even if citrate is not employed as endogenous *A. niger* iron siderophore, the fungus has a means to deal with citrate bound iron as iron source.

Utilization of Fe(III) citrate as exogenous iron siderophore complex has been shown to take place in various other microbes that do not naturally secrete citrate (Frost and Rosenberg, 1973), and of the yeasts we tested, only *C. jadinii* was unable to grow when Fe(III) citrate was added to the medium, although it grew well with Fe(II)SO_4_, and even when no iron was added to the medium. Active citrate uptake in the asexual state of *C. jadinii* (*Candida utilis*) has been shown to be subject to glucose repression (Cassio and Leao, 1991), and it could be that the utilization of glucose in our experiments prevented *C. jadinii* from being able to utilize the Fe(III) citrate complex, whereas it is able to grow on other Fe(III) salts, such as Fe(III)Cl_3_(Thomas and Dawson, 1978). This would imply that *C. jadinii* is not able to deal with Fe(III) citrate *via* RIA, which is in contrast to *S. cerevisiae* (Haas, 2014). *A. niger* utilization of the Fe(III) citrate complex did not appear to be subject to glucose repression (Table 5, Figure 6), although measurable uptake of citrate in NW186 is only observed after glucose in the medium is depleted (Supplementary File 1). The difference between uptake systems when citrate is utilized as carbon source, or complexed citrate as iron source, appears thus to be a critical aspect that warrants further investigation, but is beyond the scope of this study.

From the data presented, we cannot distinguish whether *A. niger* employs RIA or imports the entire Fe(III) citrate complex. We found a number of putative “metalloreductases with ferric-chelate reductase activity” (Supplementary File 5), but these could be specific for citrate or any of the other *A. niger* iron siderophores. However, *A. niger* citrate uptake was studied by Netik et al., who showed that, while citrate export is increased under Mn^2+^ limited conditions, import of citrate only happens when Mn^2+^ is present in the medium (Netik et al., 1997). Uptake of citrate was inhibited by EDTA, and Netik et al. hypothesized that EDTA competes for the Mn^2+^ ions. They conclude that the citrate uptake system in *A. niger* either depends on Mn^2+^ symport, or, more likely, requires the metal ion chelated form of citrate as a substrate. The requirement of Mn^2+^ ions for citrate import could be partially replaced by Mg^2+^, Fe^2+^, or Zn^2+^ (but not Cu^2+^) ions (Netik et al., 1997), indicating that the *A. niger* citrate uptake system is not necessarily restricted to the citrate-Mn^2+^ complex, but could have a broader specificity for general citrate-metal ion complexes. It is therefore likely that iron is imported as Fe(III) citrate complex, although further experimental evidence would be required to establish citrate as definite *A. niger* iron siderophore, especially in the absence of a definitively identified uptake transporter, and bearing in mind that there are other *A. niger* iron siderophores, of which only two have been identified thus far (Franken et al., 2014). Furthermore, the ability of citrate to chelate other metal ions besides iron, and the fact that citrate import had a broad specificity for general citrate-metal-ion complexes (Netik et al., 1997), could imply that *A. niger* citrate secretion is a more general mechanism to facilitate the uptake of metal ions.

### Iron and the pH-dependency of *A. niger* organic acid production

When not working with *A. niger* mutants tailored for citrate production, such as NW186, pH control is crucial to inhibit gluconate and oxalate production and enforce production of citrate instead (Ruijter et al., 1999). Certain aspects of the link between external pH and *A. niger* organic acid production have been elucidated on a molecular level. Glucose oxidase, the enzyme catalyzing the first reaction in the conversion of D-glucose to gluconate, has been shown to be stable only at pH 4-6 (Pazur and Kleppe, 1964), thus explaining the absence of gluconate at lower pH (Figure 1). The lack of oxalate at lower pH levels, on the other hand, can be explained by the finding that when the culture medium is below pH 2.5, oxalate decarboxylase, the enzyme that degrades oxalate to CO_2_ and formate (Figure 1), is synthesized (Emiliani and Bekes, 1964). In a systems level approach, Andersen et al. formulated the hypothesis that the sequential production of organic acids by *A. niger*, and specifically oxalate and citrate, leads to the most efficient acidification of the medium based on the external pH (Andersen et al., 2009). As stated by the authors, continuous acidification of the environment provides a means to effectively outcompete other organisms that are not able to thrive at a low pH, thereby providing an evolutionary advantage for the fungus (Andersen et al., 2009).

In this study, we worked with *A. niger* mutants that are incapable of producing either gluconate or both gluconate and oxalate. Therefore, it was not necessary to exert control over external culture pH to enforce citrate production. In almost all the *A. niger* strains and conditions tested, the biggest pH drop (to ~pH 2–4, Supplementary File 7) was observed after 24 h of growth. The pH continued to decrease steadily after that, albeit at a slower pace. This pattern is broken when iron is added to NW186 pre-grown without iron (Figure 2B, Supplementary File 1), or when glucose is depleted (Supplementary File 1). In both cases, the pH stops dropping and appears to even rise again (Supplementary Figures 2B, 3B in Supplementary File 1). This is likely due to consumption of citrate in the glucose depleted cultures, but it is not clear what the fungus is taking back up in the case of the iron pulse. Although it is tempting to link the rising pH to the net uptake of an iron-citrate complex, it is also possible that the pH rises due to an increased activity of H^+^ symport of another iron-siderophore or compound.

A general observation in fungi is that organic acid secretion is actually higher at higher external pH, and that there is a continuous influx and efflux of organic acids (Vrabl et al., 2012). Besides the discussed hypothesis that acidification of the medium might serve to outcompete other organisms, acidification of the environment is also a means of increasing iron solubility and thus bioavailability (Dutton and Evans, 1996; Gadd, 1999). Based on the results presented and discussed, we propose that citrate, and possibly also oxalate and gluconate, are not just secreted to acidify the medium, but that the sequential secretion of gluconate, oxalate, and then citrate is based on optimally increasing bioavailability of iron based on their own chelating properties at the given external pH, and as such serve as *A. niger* iron siderophores. The fact that oxalate secretion precedes citrate secretion until pH ≤ 2.5 can be associated to the lower stability constant of the Fe(III) oxalate complex compared to the Fe(III) citrate complex [9.4 compared to 11.85 (Furia, 2006)], implying that the Fe(III) citrate complex is more stable at lower pH values.

The increased correlation between iron limitation and citrate production observed in the exclusively citrate producing *A. niger* mutant NW186, even at a pH that would usually be considered suboptimal for citrate production (Ruijter et al., 1999), is due to citrate being the only organic acid available for a task that would otherwise be shared between, and optimally adjusted to, multiple organic acids. The dependency of *A. niger* organic acid production on ambient pH also draws parallels to iron siderophore metabolism in *Aspergillus nidulans*, where, consistent with the insolubility of iron at alkaline pH, production of *A. nidulans* iron siderophores increases with an increase in culture pH (Eisendle et al., 2004).

Based on these insights, our findings, and in line with the observation that *A. niger* imports citrate only as metal-ion complex (Netik et al., 1997), we suggests that increased citrate secretion under iron limited conditions is a physiological response to, rather than just a consequence of, low iron availability. Specifically, we propose that the reason *A. niger* citrate synthesis is actively up-regulated under iron limited conditions is because the fungus employs citrate as iron siderophore.

## Author contributions

DO, TS, VM, MS, and PS conceived and designed the work. MG and TS performed the experiments. DO, TS, and MS analyzed the data. DO, MG, TS, JT, MS, and PS contributed to the interpretation of the data. DO wrote the manuscript, and MS and PS participated therein. DO, MG, TS, JT, VM, MS, and PS critically revised the manuscript for intellectual content. All authors have read and agree to the submission of the manuscript.

### Conflict of interest statement

The authors declare that the research was conducted in the absence of any commercial or financial relationships that could be construed as a potential conflict of interest.

## References

[B1] Andersen M. R. Lehmann L. Nielsen J. (2009). Systemic analysis of the response of *Aspergillus niger* to ambient pH. Genome Biol. 10:R47. 10.1186/gb-2009-10-5-r47 19409083 PMC2718513

[B2] Andersen M. R. Salazar M. P. Schaap P. J. Van De Vondervoort P. J. I. Culley D. Thykaer J.. (2011). Comparative genomics of citric-acid-producing *Aspergillus niger* ATCC 1015 versus enzyme-producing CBS 513.88. Genome Res. 21, 885–897. 10.1101/gr.112169.110 21543515 PMC3106321

[B3] Blatzer M. Barker B. M. Willger S. D. Beckmann N. Blosser S. J. Cornish E. J.. (2011). SREBP coordinates iron and ergosterol homeostasis to mediate triazole drug and hypoxia responses in the human fungal pathogen *Aspergillus fumigatus*. PLoS Genet. 7:e1002374. 10.1371/journal.pgen.1002374 22144905 PMC3228822

[B4] Bolger A. M. Lohse M. Usadel B. (2014). Trimmomatic: a flexible trimmer for Illumina sequence data. Bioinformatics 30, 2114–2120. 10.1093/bioinformatics/btu170 24695404 PMC4103590

[B5] Cassio F. Leao C. (1991). Low- and high-affinity transport systems for citric acid in the yeast *Candida utilis*. Appl. Environ. Microbiol. 57, 3623–3628. 1664712 10.1128/aem.57.12.3623-3628.1991 PMC184023

[B6] Chesters C. G. Rolinson G. N. (1951). Zinc in the metabolism of a strain of *Aspergillus niger*. J. Gen. Microbiol. 5, 553–558. 10.1099/00221287-5-3-553 14873901

[B7] Claudel-Renard C. Chevalet C. Faraut T. Kahn D. (2003). Enzyme-specific profiles for genome annotation: PRIAM. Nucleic Acids Res. 31, 6633–6639. 10.1093/nar/gkg847 14602924 PMC275543

[B8] Cox C. D. (1980). Iron uptake with ferripyochelin and ferric citrate by *Pseudomonas aeruginosa*. J. Bacteriol. 142, 581–587. 6769903 10.1128/jb.142.2.581-587.1980 PMC294027

[B9] Cox G. B. Gibson F. Luke R. K. Newton N. A. O'Brien I. G. Rosenberg H. (1970). Mutations affecting iron transport in *Escherichia coli*. J. Bacteriol. 104, 219–226. 4919746 10.1128/jb.104.1.219-226.1970 PMC248203

[B10] Currie J. N. (1917). The citric acid fermentation of *Aspergillus niger*. J. Biol. Chem. 31, 15–37.

[B11] Dobin A. Davis C. A. Schlesinger F. Drenkow J. Zaleski C. Jha S.. (2013). STAR: ultrafast universal RNA-seq aligner. Bioinformatics 29, 15–21. 10.1093/bioinformatics/bts635 23104886 PMC3530905

[B12] Dutton M. V. Evans C. S. (1996). Oxalate production by fungi: its role in pathogenicity and ecology in the soil environment. Can. J. Microbiol. 42, 881–895. 10.1139/m96-114

[B13] Eisendle M. Oberegger H. Buttinger R. Illmer P. Haas H. (2004). Biosynthesis and uptake of siderophores is controlled by the PacC-mediated ambient-pH regulatory system in *Aspergillus nidulans*. Eukaryotic Cell 3, 561–563. 10.1128/EC.3.2.561-563.2004 15075286 PMC387658

[B14] Emiliani E. Bekes P. (1964). Enzymatic oxalate decarboxylation in *Aspergillus niger*. Arch. Biochem. Biophys. 105, 488–493. 10.1016/0003-9861(64)90040-2 14236631

[B15] Franken A. C. W. Lechner B. E. Werner E. R. Haas H. Lokman B. C. Ram A. F. J.. (2014). Genome mining and functional genomics for siderophore production in *Aspergillus niger*. Brief. Funct. Genomics 13, 482–492. 10.1093/bfgp/elu026 25062661

[B16] Frost G. E. Rosenberg H. (1973). The inducible citrate-dependent iron transport system in *Escherichia coli* K12. Elsevier 330, 90–101. 10.1016/0005-2736(73)90287-3 4587079

[B17] Furia T. E. (2006). Stability Constants (log K1) of Various Metal Chelates, in Sequestrants in Foods, 2 Available online at: http://www.coldcure.com/html/stability_constants.html

[B18] Gadd G. M. (1999). Fungal production of citric and oxalic acid: importance in metal speciation, physiology and biogeochemical processes. Adv. Microb. Physiol. 41, 47–92. 10.1016/S0065-2911(08)60165-4 10500844

[B19] Goosen T. van Engelenburg F. Debets F. Swart K. Bos K. van den Broek H. (1989). Tryptophan auxotrophic mutants in *Aspergillus niger*: inactivation of the trpC gene by cotransformation mutagenesis. Mol. Gen. Genet. 219, 282–288. 10.1007/BF00261189 2615762

[B20] Gründlinger M. Yasmin S. Lechner B. E. Geley S. Schrettl M. Hynes M.. (2013). Fungal siderophore biosynthesis is partially localized in peroxisomes. Mol. Microbiol. 88, 862–875. 10.1111/mmi.12225 23617799 PMC3709128

[B21] Guerinot M. L. Yi Y. (1994). Iron: nutritious, noxious, and not readily available. Plant Physiol. 104, 815–820. 10.1104/pp.104.3.815 12232127 PMC160677

[B22] Guerinot M. L. Meidl E. J. Plessner O. (1990). Citrate as a Siderophore in *Bradyrhizobium japonicum*. J. Bacteriol. 172, 3298–3303. 10.1128/jb.172.6.3298-3303.1990 2140566 PMC209139

[B23] Haas H. (2003). Molecular genetics of fungal siderophore biosynthesis and uptake: the role of siderophores in iron uptake and storage. Appl. Microbiol. Biotechnol. 62, 316–330. 10.1007/s00253-003-1335-2 12759789

[B24] Haas H. (2012). Iron - a key nexus in the virulence of *Aspergillus fumigatus*. Front. Microbiol. 3:28. 10.3389/fmicb.2012.00028 22347220 PMC3272694

[B25] Haas H. (2014). Fungal siderophore metabolism with a focus on *Aspergillus fumigatus*. Nat. Prod. Rep. 31, 1266–1276. 10.1039/C4NP00071D 25140791 PMC4162504

[B26] Han Y. Joosten H. J. Niu W. Zhao Z. Mariano P. S. McCalman M.. (2007). Oxaloacetate hydrolase, the C-C bond lyase of oxalate secreting fungi. J. Biol. Chem. 282, 9581–9590. 10.1074/jbc.M608961200 17244616

[B27] Hortschansky P. Eisendle M. Al-Abdallah Q. Schmidt A. D. Bergmann S. Thön M.. (2007). Interaction of HapX with the CCAAT-binding complex—a novel mechanism of gene regulation by iron. EMBO J. 26, 3157–3168. 10.1038/sj.emboj.7601752 17568774 PMC1914100

[B28] Jaklitsch W. M. Kubicek C. P. Scrutton M. C. (1991). Intracellular location of enzymes involved in citrate production by *Aspergillus niger*. Can. J. Microbiol. 37, 823–827. 10.1139/m91-142 1777859

[B29] Kanehisa M. Goto S. (2000). KEGG: kyoto encyclopedia of genes and genomes. Nucleic Acids Res. 28, 27–30. 10.1093/nar/28.1.27 10592173 PMC102409

[B30] Kanehisa M. Sato Y. Kawashima M. Furumichi M. Tanabe M. (2015). KEGG as a reference resource for gene and protein annotation. Nucleic Acids Res. 44, D457–D462. 10.1093/nar/gkv1070 26476454 PMC4702792

[B31] Karaffa L. Kubicek C. P. (2003). *Aspergillus niger* citric acid accumulation: do we understand this well working black box? Appl. Microbiol. Biotechnol. 61, 189–196. 10.1007/s00253-002-1201-7 12698275

[B32] Kopylova E. Noé L. Touzet H. (2012). SortMeRNA: fast and accurate filtering of ribosomal RNAs in metatranscriptomic data. Bioinformatics 28, 3211–3217. 10.1093/bioinformatics/bts611 23071270

[B33] Kubicek C. P. Röhr M. (1977). Influence of manganese on enzyme synthesis and citric acid accumulation in *Aspergillus niger*. Eur. J. Appl. Microbiol. 4, 167–175. 10.1007/BF01390476

[B34] Kubicek C. P. Röhr M. (1978). The role of the tricarboxylic acid cycle in citric acid accumulation by *Aspergillus niger*. Eur. J. Appl. Microbiol. Biotechnol. 5, 263–271. 10.1007/BF00504714

[B35] Kubicek C. P. Röhr M. (1985). Aconitase and citric acid fermentation by *Aspergillus niger*. Appl. Environ. Microbiol. 50, 1336–1338. 4091562 10.1128/aem.50.5.1336-1338.1985 PMC238755

[B36] Kubicek C. P. Schreferl-Kunar G. Wöhrer W. Röhr M. (1988). Evidence for a cytoplasmic pathway of oxalate biosynthesis in *Aspergillus niger*. Appl. Environ. Microbiol. 54, 633–637. 3132096 10.1128/aem.54.3.633-637.1988 PMC202517

[B37] Kusters-van Someren M. A. Harmsen J. A. Kester H. C. Visser J. (1991). Structure of the *Aspergillus niger* pelA gene and its expression in *Aspergillus niger* and *Aspergillus nidulans*. Curr. Genet. 20, 293–299. 10.1007/BF00318518 1934134

[B38] La Nauze J. M. (1966). Aconitase and isocitric dehydrogenases of *Aspergillus niger* in relation to citric acid production. J. Gen. Microbiol. 44, 73–81. 10.1099/00221287-44-1-73 5965361

[B39] Meijer S. Nielsen M. L. Olsson L. Nielsen J. (2009). Gene deletion of cytosolic ATP:citrate lyase leads to altered organic acid production in *Aspergillus niger*. J. Ind. Microbiol. Biotechnol. 36, 1275–1280. 10.1007/s10295-009-0607-y 19554356

[B40] Meixner-Monori B. Kubicek C. P. Habison A. Kubicek-Pranz E. M. Röhr M. (1985). Presence and regulation of the alpha-ketoglutarate dehydrogenase multienzyme complex in the filamentous fungus *Aspergillus niger*. J. Bacteriol. 161, 265–271. 3968029 10.1128/jb.161.1.265-271.1985 PMC214866

[B41] Neilands J. B. (1981). Microbial iron compounds. Annu. Rev. Biochem. 50, 715–730. 10.1146/annurev.bi.50.070181.003435 6455965

[B42] Neilands J. B. (1995). Siderophores: structure and function of microbial iron transport compounds. J. Biol. Chem. 270, 26723–26726. 10.1074/jbc.270.45.26723 7592901

[B43] Netik A. Torres N. V. Riol J. M. Kubicek C. P. (1997). Uptake and export of citric acid by *Aspergillus niger* is reciprocally regulated by manganese ions. Biochim. Biophys. Acta Biomembr. 1326, 287–294. 10.1016/S0005-2736(97)00032-1 9218559

[B44] Nordberg H. Cantor M. Dusheyko S. Hua S. Poliakov A. Shabalov I.. (2014). The genome portal of the department of energy joint genome institute: 2014 updates. Nucleic Acids Res. 42, D26–D31. 10.1093/nar/gkt1069 24225321 PMC3965075

[B45] Pazur J. H. Kleppe K. (1964). The oxidation of glucose and related compounds by glucose oxidase from *Aspergillus niger*. Biochemsitry 3, 578–583. 10.1021/bi00892a018 14188176

[B46] Quinlan A. R. Hall I. M. (2010). BEDTools: a flexible suite of utilities for comparing genomic features. Bioinformatics 26, 841–842. 10.1093/bioinformatics/btq033 20110278 PMC2832824

[B47] R Core T. (2014). R: A Language and Environment for Statistical Computing. Available online at: http://www.r-project.org/

[B48] Ratledge C. (2000). Look before you clone. FEMS Microbiol. Lett. 189, 317–318. 10.1111/j.1574-6968.2000.tb09250.x 10991634

[B49] Ratledge C. Ruijter G. J. G. (2000). Life is not that simple. FEMS Microbiol. Lett. 189, 317–318. 10991634 10.1111/j.1574-6968.2000.tb09250.x

[B50] Robinson M. D. McCarthy D. J. Smyth G. K. (2009). edgeR: a Bioconductor package for differential expression analysis of digital gene expression data. Bioinformatics 26, 139–140. 10.1093/bioinformatics/btp616 19910308 PMC2796818

[B51] Ruijter G. J. G. Bax M. Patel H. Flitter S. J. Van De Vondervoort P. J. I. De Vries R. P.. (2003). Mannitol is required for stress tolerance in *Aspergillus niger* conidiospores. Eukaryotic Cell 2, 690–698. 10.1128/EC.2.4.690-698.2003 12912888 PMC178341

[B52] Ruijter G. J. G. Panneman H. Visser J. (1997). Overexpression of phosphofructokinase and pyruvate kinase in citric acid-producing *Aspergillus niger*. Biochim. Biophys. Acta Gen. Subj. 1334, 317–326. 10.1016/S0304-4165(96)00110-9 9101728

[B53] Ruijter G. J. G. Panneman H. Xu D. B. Visser J. (2000). Properties of *Aspergillus niger* citrate synthase and effects of *citA* overexpression on citric acid production. FEMS Microbiol. Lett. 184, 35–40. 10.1111/j.1574-6968.2000.tb08986.x 10689162

[B54] Ruijter G. J. G. Van De Vondervoort P. J. I. Visser J. (1999). Oxalic acid production by *Aspergillus niger*: an oxalate-non-producing mutant produces citric acid at pH 5 and in the presence of manganese. Microbiology 145, 2569–2576. 10.1099/00221287-145-9-2569 10517610

[B55] Schomburg I. Chang A. Ebeling C. Gremse M. Heldt C. Huhn G.. (2004). BRENDA, the enzyme database: updates and major new developments. Nucleic Acids Res. 32, D431–D433. 10.1093/nar/gkh081 14681450 PMC308815

[B56] Schrader M. Fahimi H. D. (2006). Peroxisomes and oxidative stress. Biochim. Biophys. Acta Mol. Cell Res. 1763, 1755–1766. 10.1016/j.bbamcr.2006.09.006 17034877

[B57] Schrettl M. Kim H. S. Eisendle M. Kragl C. Nierman W. C. Heinekamp T.. (2008). SreA-mediated iron regulation in *Aspergillus fumigatus*. Mol. Microbiol. 70, 27–43. 10.1111/j.1365-2958.2008.06376.x 18721228 PMC2610380

[B58] Schuster E. Dunn-Coleman N. Frisvad J. Van Dijck P. (2002). On the safety of *Aspergillus niger* A review. Appl. Microbiol. Biotechnol. 59, 426–435. 10.1007/s00253-002-1032-6 12172605

[B59] Silva A. M. N. Kong X. Parkin M. C. Cammack R. Hider R. C. (2009). Iron(III) citrate speciation in aqueous solution. Dalton Trans. 8616–8625. 10.1039/b910970f 19809738

[B60] Szczodrak J. Ilczuk Z. (1985). Effect of iron on the activity of aconitate hydratase and synthesis of citric acid by *Aspergillus niger*. Zentralbl. Mikrobiol. 140, 567–574. 4090766

[B61] Thomas K. C. Dawson P. S. S. (1978). Relationship between iron-limited growth and energy limitation during phased cultivation of *Candida utilis*. Can. J. Microbiol. 24, 440–447. 10.1139/m78-073 565248

[B62] Verduyn C. Postma E. Scheffers W. A. Van Dijken J. P. (1992). Effect of benzoic acid on metabolic fluxes in yeasts: a continuous-culture study on the regulation of respiration and alcoholic fermentation. Yeast 8, 501–517. 10.1002/yea.320080703 1523884

[B63] Vishniac W. Santer M. (1957). The Thiobacilli. Bacteriol. Rev. 21, 195–213. 13471458 10.1128/br.21.3.195-213.1957 PMC180898

[B64] Vrabl P. Fuchs V. Pichler B. Schinagl C. W. Burgstaller W. (2012). Organic acid excretion in *Penicillium ochrochloron* increases with ambient pH. Front. Microbiol. 3:121. 10.3389/fmicb.2012.00121 22493592 PMC3318189

[B65] Yin X. Shin H.-D. Li J. Du G. Liu L. Chen J. (2017). Comparative genomics and transcriptome analysis of *Aspergillus niger* and metabolic engineering for citrate production. Sci. Rep. 7:41040. 10.1038/srep41040 28106122 PMC5247736

