# Supplementary material for: Aspergillus niger Secretes Citrate to Increase Iron Bioavailability
Source: Front Microbiol. 2017 Aug 2;8:1424. doi: 10.3389/fmicb.2017.01424 (PMC5539119; doi:10.3389/fmicb.2017.01424)
Supplement: Supplementary file 1 [file DataSheet1.DOCX]

Supplementary Material

*Aspergillus niger* secretes citrate to increase iron bioavailability

Dorett I Odoni, Merlijn P van Gaal, Tom Schonewille, Juan A Tamayo-Ramos, Vitor A P Martins dos Santos, Maria Suarez-Diez and Peter J Schaap^*^

*** Correspondence:** Corresponding Author: peter.schaap@wur.nl

To exclude that our observations are due to the transformation marker (*ΔargB*) in NW305 and NW186, we measured citrate per glucose production of an alternative, gluconate non-producing strain, NW129 (*cspA1, goxC17, pyrA1*). NW129 and NW305 have the same genotype except for their transformation markers, making them auxotrophic for arginine (Δ*argB*) or uridine (*pyrA1*), respectively.


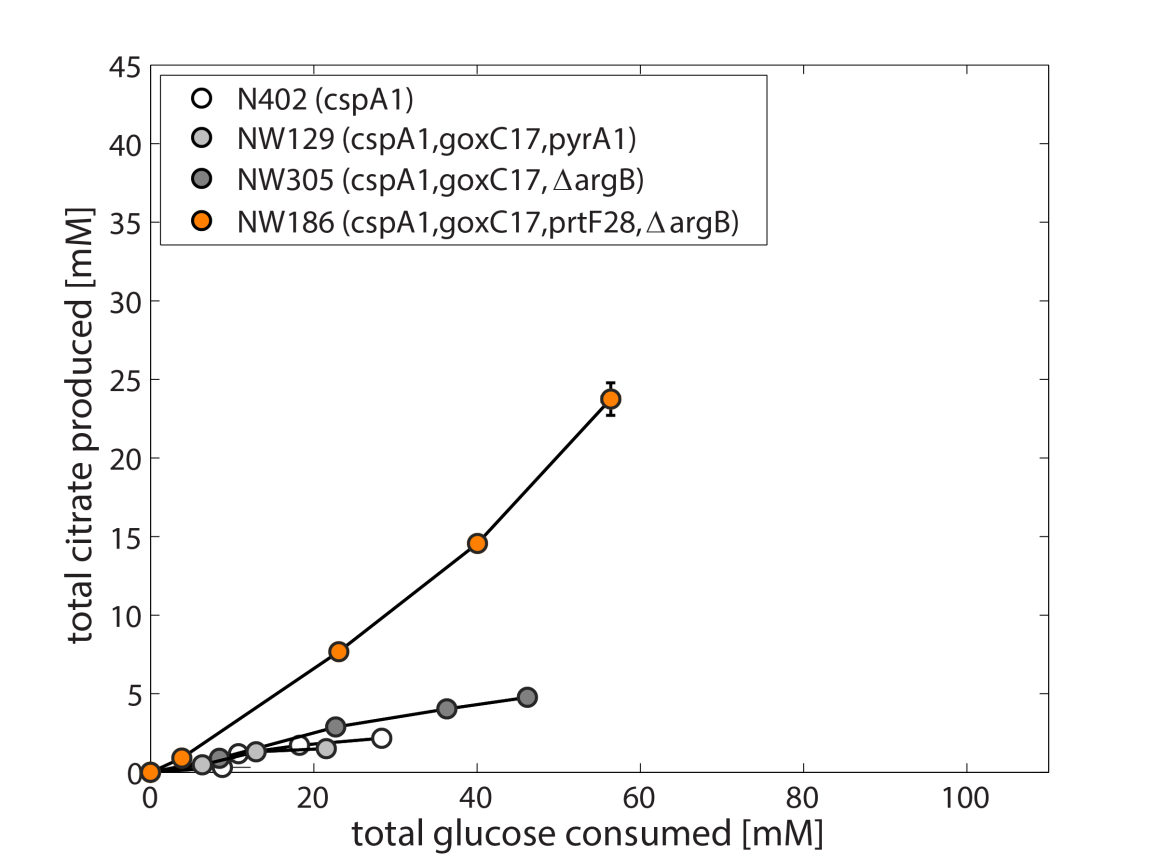


**Supplementary Figure 1.** Total citrate production per glucose consumption of *A. niger* N402 and its derivatives under iron limited conditions (no Fe added to the medium). Measurement points were taken once every 24 h and show the average of two biological replicates.

We found that NW305 and NW129 do not behave differently under iron limited conditions with regard to the amount of citrate produced per glucose consumed, especially in comparison to NW186 (Supplementary Figure 1). As the difference between *A. niger* NW305 and NW186 is the lack of functioning oxalate biosynthesis *via* OahA, we conclude that increased citrate production under low iron stress is a result of impaired oxalate biosynthesis, and not the Δ*argB* mutation.

**
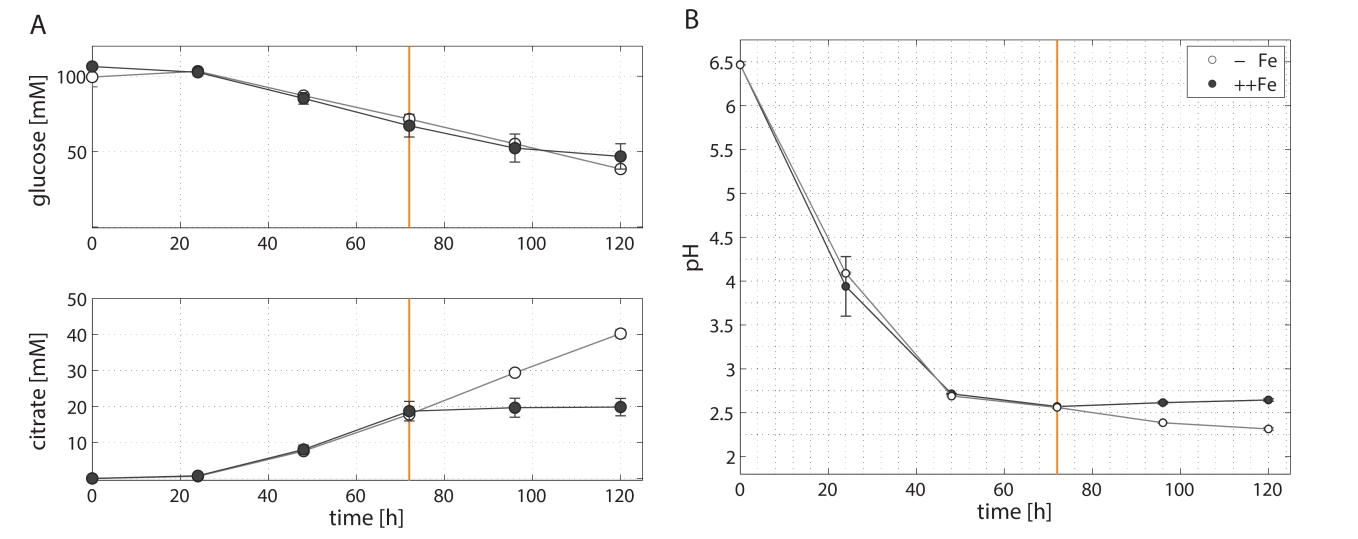
**

**Supplementary Figure 2.** Total glucose and citrate concentration (A), and medium acidification (B) of *A. niger* NW186 pre-grown without iron in the medium, and with (filled symbols) or without (empty symbols) Fe(II)SO_4_ added to the medium at t = 72 h (orange line). Measurement points were taken once every 24 h and show the average of two biological replicates

**
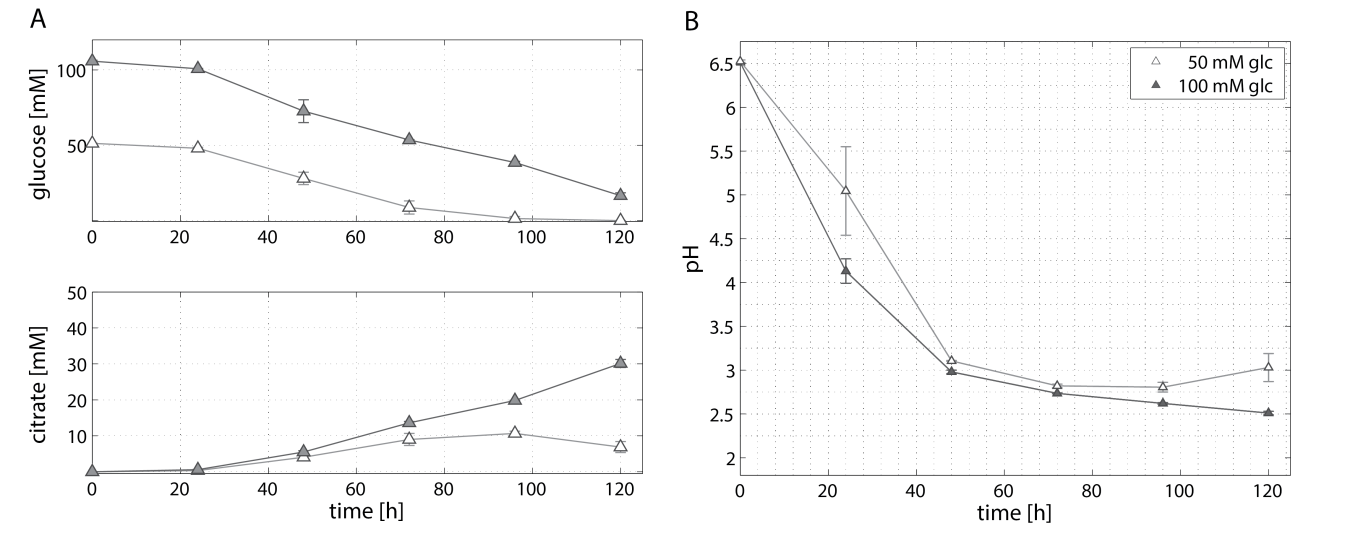
**

**Supplementary Figure 3.** Total glucose and citrate concentration (A), and medium acidification (B) of *A. niger* NW186 grown with 100 mM (filled symbols) or 50 mM (empty symbols) glucose. Measurement points were taken once every 24 h and show the average of two biological replicates.
