# Supplementary material for: Aspergillus niger Secretes Citrate to Increase Iron Bioavailability
Source: Front Microbiol. 2017 Aug 2;8:1424. doi: 10.3389/fmicb.2017.01424 (PMC5539119; doi:10.3389/fmicb.2017.01424)
Supplement: Supplementary file 2 [file DataSheet2.DOCX]

Supplementary Material

*Aspergillus niger* secretes citrate to increase iron bioavailability

Dorett I Odoni, Merlijn P van Gaal, Tom Schonewille, Juan A Tamayo-Ramos, Vitor A P Martins dos Santos, Maria Suarez-Diez and Peter J Schaap^*^

*** Correspondence:** Corresponding Author: peter.schaap@wur.nl

In the following, we have provided one **example** command for every tool used for the RNA seq data processing.

Filtering: ================================================================= 1) get rid of rRNA (.fastq and .log are added by default): .-----------------------------------------------------

Tool: SortMeRNA v1.9 -note that that database files have to be obtained separately.

user$ <user_directory>/sortmerna-1.9-linux-64-bin/sortmerna -n 9 --db <user_directory>/sortmerna-1.9-linux-64-bin/rRNA_databases/rfam-5.8s-database-id98.fasta <user_directory>/sortmerna-1.9-linux-64-bin/rRNA_databases/rfam-5s-database-id98.fasta <user_directory>/sortmerna-1.9-linux-64-bin/rRNA_databases/silva-arc-16s-database-id95.fasta <user_directory>/sortmerna-1.9-linux-64-bin/rRNA_databases/silva-arc-23s-database-id98.fasta <user_directory>/sortmerna-1.9-linux-64-bin/rRNA_databases/silva-bac-16s-database-id85.fasta <user_directory>/sortmerna-1.9-linux-64-bin/rRNA_databases/silva-bac-23s-database-id98.fasta <user_directory>/sortmerna-1.9-linux-64-bin/rRNA_databases/silva-euk-18s-database-id95.fasta <user_directory>/sortmerna-1.9-linux-64-bin/rRNA_databases/silva-euk-28s-database-id98.fasta <user_directory>/filtering/sortmerna-1.9-linux-64-bin/rRNA_databases/coliphage_phi-X174.fasta -a 6 –I <user_directory>/1_G9_305_0Fe__2_T48_13391_GATCAG_L001_R1_001_BHWMYMADXX.filt.fastq --accept <user_directory>/305w0xFe_1_T48_filt_rRNA --other <user_directory>/305w0xFe_1_T48_filt_mRNA –log <user_directory>/305w0xFe_1_T48_rRNAfiltering

- repeat for the all the datasets.

2) get rid of adapters: -----------------------------------------------------------------------------------------------

Tool: Trimmomatic v0.32

user$ java -classpath <user_directory>/Trimmomatic-0.32/trimmomatic-0.32.jar org.usadellab.trimmomatic.TrimmomaticSE -threads 4 -trimlog <user_directory>/NW305w0xFe_1_T48_trimmomatic.log <user_directory>/305w0xFe_1_T48_filt_mRNA.fastq <user_directory>/305w0xFe_1_T48_filt_mRNA_trimmomatic.fastq ILLUMINACLIP: <user_directory>/Trimmomatic-0.32/adapters/all_adapters.fa:2:30:10 MINLEN:50 LEADING:3 TRAILING:3 SLIDINGWINDOW:4:15

- repeat for the all the datasets.

End filtering. ==============================================================

Mapping the QC sequence reads: ================================================

1) Indexing the reference file (redo): .---------------------------------------------------------------------------

Tool: STAR v2.5.0c

user$ <user_directory>/STAR-2.5.0c/bin/Linux_x86_64/STAR --genomeDir <user_directory>/Aniger_ATCC1015_CDS/ --runMode genomeGenerate --genomeFastaFiles <user_directory>/Aspni7_GeneCatalog_CDS_20131226.fasta --runThreadN 20

2) Doing the mapping: ----------------------------------------------------------------------------------------------

user$ <user_directory>/STAR-2.5.0c/bin/Linux_x86_64/STAR --genomeDir <user_directory>/Aniger_ATCC1015/ --readFilesIn <user_directory>/305w0xFe_1_T48_filt_mRNA_trimmomatic.fastq --outFileNamePrefix <user_directory>/NW305_0xFe/1/ --runThreadN 20

- repeat for the all the datasets.

3) Converting the .sam file to a .bam file, then sort and index the file: --------------------------------------

user$ samtools view -b -S <user_directory>/NW305_0xFe/1/Aligned.out.sam -o <user_directory>/NW305_0xFe_1_filtmRNAtrimmomatic_ATCC1015_CDS_aligned.bam

user$ samtools sort <user_directory>/NW305_0xFe_1_filtmRNAtrimmomatic_ATCC1015_CDS_aligned.bam <user_directory>/NW305_0xFe_1_filtmRNAtrimmomatic_ATCC1015_CDS_aligned_sorted

user$ samtools index <user_directory>/NW305_0xFe_1_filtmRNAtrimmomatic_ATCC1015_CDS_aligned_sorted.bam

- repeat for the all the datasets.

End mapping ==============================================================

The aligned .bam files were deposited at the European Nucleotide Archive (ENA) under the accession number PRJEB20746 (http://www.ebi.ac.uk/ena/data/view/ PRJEB20746).

Expression levels ===========================================================

Average nucleotide coverage calculation (only for rough estimation): --------------------------------------

1) Prepare the genome: ...……….…………………………………………………………………….... Required is a tab-separated input file with the following format: geneId’\t’length’\n’

This can be generated from an input fasta file with the following Python script: .*****************

Input arguments: argv1 = inputfile.fa, argv2 = outputfile_genome.txt

user$ python <user_directory>/<script>.py <user_directory>/<database>.fasta <user_directory>/<outputfile>_genome.txt

Start script ************************************************************************

# reads an input .fasta file and makes a tab delimited genome file with: contig+'\t'+length+'\n'

# sys.argv[1] = input file name

# sys.argv[2] = output file name

import sys

import re

# open the in- and output files:

sourceFile = open(sys.argv[1],'r')

destFile = open(sys.argv[2],'w')

# read the input file and add an empty line at the end:

sourceLines = (open(sys.argv[1],'r')).readlines()

sourceLines += ['>\n']

# inititate variables:

length = 0

GOannot = False

# get the information from the input file and write it to the output file:

for line in sourceLines:

sline = line.strip()

if not re.search(r'>',sline) and GOannot == True:

for ch in sline:

length += 1

if sline.startswith('>') and GOannot == True:

destFile.write(contig[1:]+'\t'+str(length)+'\n')

GOannot = False

length = 0

if sline.startswith('>'):

GOannot = True

contig = sline[1:] # take the whole header

# close the in- and output files:

sourceFile.close()

destFile.close()

End script .************************************************************************

2) Actual coverage calculation: ...............................................................................................................

Tool: BEDTools v2.17.0

Naming outputfile: <seqs>_<reference>_bedCoverage.txt

user$ <user_directory>/bedtools-2.17.0/bin/genomeCoverageBed -d -ibam <user_directory>/ NW305_0xFe_1_filtmRNAtrimmomatic_ATCC1015_CDS_aligned_sorted.bam -g <user_directory>/ ATCC1015CDS_genome.txt > <user_directory>/ N402ox0xFe1T48filtmRNAtrimmomatic_AnigerATCC1015CDS_bedCoverage.txt

- repeat for the all the datasets.

The average nucleotide coverage per gene can be obtained with the following Python script: *******

Input arguments: argv1 = inputfile_bedCoverage.txt, argv2 = outputfile_bedCoverage_av.txt, argv3 = name of the first contig in the file (e.g. jgi\|Aspni7\|1130422\|fgenesh1_pg.chr_101_#_1).

user$ python <user_directory>/<script>.py <user_directory>/N402ox0xFe1T48filtmRNAtrimmomatic_AnigerATCC1015CDS_bedCoverage.txt <user_directory>/N402ox0xFe1T48filtmRNAtrimmomatic_AnigerATCC1015CDS_bedCoverage_av.txt jgi\|Aspni7\|1130422\|fgenesh1_pg.chr_101_#_1

- repeat for all the datasets.

Start script ************************************************************************

# reads _bedCoverage.txt input file created with genomeCoverageBed and makes a tab delimited file # that gives the average nucleotide coverage per contig.

# sys.argv[1] = input file name

# sys.argv[2] = output file name

# sys.argv[3] = first geneId in input file

import sys

# open the in- and output files:

sourceFile = open(sys.argv[1],'r')

destFile = open(sys.argv[2],'w')

# initiate variables

contigName = str(sys.argv[3])

covList = []

c = 0

covListSum = 0

# get the information from the input file:

for line in sourceFile.readlines():

sline = line.strip()

spline = sline.split('\t')

if spline[0] != contigName:

for elem in covList:

c += 1

covListSum += float(elem)

covListAv = covListSum/c

destFile.write(contigName+'\t'+str(covListAv)+'\n')

c = 0

covListSum = 0

contigName = spline[0]

covList = []

covList += [spline[2]]

else:

covList += [spline[2]]

# write the information to the output file:

for elem in covList:

c += 1

covListSum += float(elem)

covListAv = covListSum/c

destFile.write(contigName+'\t'+str(covListAv)+'\n')

# close the in- and output files:

sourceFile.close()

destFile.close()

End script .************************************************************************

As an extra, we corrected for inequal sequencing depth by calculating the sum of the average nucleotide coverages for each dataset, and dividing the averages by a normalisation factor based on the smallest dataset.

End expression levels .========================================================

Differential expression analysis (pre-files) .=========================================

The input for differential expression analysis with edgeR is a file containing read counts per reference transcript. To obtain the read count file, a reference .gff file that matches with the .bam file mapping is required (not provided here).

Tool: BEDTools v2.17.0

user$ <user_directory>/bedtools-2.17.0/bin/coverageBed -abam <user_directory>/ NW305_0xFe_1_filtmRNAtrimmomatic_ATCC1015_CDS_aligned.bam -b <user_directory>/<file_matching_reference_used_for_mapping>.gff > <user_directory>/ NW305_0xFe_1_filtmRNAtrimmomatic_ATCC1015CDS_readCount

- repeat for all the datasets, and convert to files that contain the exon specific read count information, as described in the edgeR user manual (Robinson, McCarthy, and Smyth 2010). The differential expression analysis itself was also performed as desribed in the user manual (see below).

Robinson, M. D., D. J. McCarthy, and G. K. Smyth. 2010. “edgeR: A Bioconductor Package for Differential Expression Analysis of Digital Gene Expression Data.” *Bioinformatics* 26 (1). Oxford University Press: 139–40. doi:10.1093/bioinformatics/btp616.

End pre-files differential expression analysis ========================================

Differential expression analysis (in R) .============================================

Install edgeR --------------------------------------------------------------------------------------------------------- *********************************************************************************

source("https://bioconductor.org/biocLite.R")

biocLite("edgeR")

biocLite("locfit")

biocLite("statmod")

*********************************************************************************

General ....................................................................................................................................................

# load the package:

library(edgeR)

# set the work directory:

setwd("<user_directory>")

NW305 -Fe *vs* NW305 ++Fe ………………………………...................................................................

# read the counts from the file containing the exon specific read count information (see edgeR user manual):

x <- read.delim("NW305w0xFe10xFe_ATCC1015CDS_readCount_transId_edgeRinput",row.names="Symbol")

# indicate which samples are biological replicates:

group <- factor(c(1,1,2,2))

# create the edgeR object for differential expression analysis:

y <- DGEList(counts=x,group=group)

y ...................................................................................................

$samples

group lib.size norm.factors

c1 1 36845596 1

c2 1 43055450 1

c3 2 33795444 1

c4 2 20003815 1

.....................................................................................................

# filtering and normalisation (discard very lowly expressed genes, as they are not biologically relevant and will mess with the statistics later on. However, keep lowly expressed genes if they are only expressed in one of the two conditions). Do this on counts per million to account for library sizes:

keep <- rowSums(cpm(y)>1) >= 2 # in at least 2 samples

y <- y[keep, ,keep.lib.sizes=FALSE]

y ...................................................................................................

$samples

group lib.size norm.factors

c1 1 36823141 1

c2 1 43034700 1

c3 2 33765940 1

c4 2 19989426 1

.....................................................................................................

# calculate the normalisation factor to account for different library sizes:

y <- calcNormFactors(y) # default: TMM

y ...................................................................................................

$samples

group lib.size norm.factors

c1 1 36823141 0.9640047

c2 1 43034700 0.8980912

c3 2 33765940 1.0905633

c4 2 19989426 1.0591306

.....................................................................................................

# Define the design matrix based on the experimental design:

design <- model.matrix(~group)

# estimate the dispersion:

y <- estimateDisp(y,design,robust=TRUE)

y$common.dispersion

[1] 0.02334323

# test for DE genes (exact test is applicable to experiments with a single factor):

et <- exactTest(y,pair=c(1,2))

topTags(et) # just for own information

# write the output to a file (make n high so that all is included):

write.table(topTags(et,n=50000,sort.by="logFC",adjust.method="BH",p.value=0.05),file="NW305w0xFe10xFe_ATCC1015CDS_readCount_transId_edgeRoutput_temp",quote=FALSE,sep="\t",row.names=TRUE,col.names=TRUE)

The above testing method identifies differential expression based on statistical significance, regardless of how small the difference might be. The values could be sorted based on p-value, and then only values with log2FC > 1 (for 2-fold) or > 0.58 (for 1.5-fold) could be selected, but this would favour lowly expressed but highly variable genes (according to edgeR manual). Therefore, we applied (log-fold-change) testing. We used a threshold of 0.58 (the fold change, 1.5 in this case, below which we are definitely not interested in the gene). This is justified for eukaryotes.

fit <- glmFit(y,design)

tr <- glmTreat(fit,coef=2,lfc=0.58)

topTags(tr) # for own information

# write the output to a file (make n high so that all is included):

write.table(topTags(tr,n=50000,sort.by="logFC",adjust.method="BH",p.value=0.05),file="NW305w0xFe10xFe_ATCC1015CDS_readCount_transId_edgeRoutput",quote=FALSE,sep="\t",row.names=TRUE,col.names=TRUE)

write.table(topTags(tr,n=50000,sort.by="logFC",adjust.method="BH"),file="NW305w0xFe10xFe_ATCC1015CDS_readCount_transId_edgeRoutput_noCutoff",quote=FALSE,sep="\t",row.names=TRUE,col.names=TRUE)

The same procedure was repeated for NW305 -Fe *vs* NW186 -Fe.

End differential expression analysis ==============================================
