# Supplementary material for: Aspergillus niger Secretes Citrate to Increase Iron Bioavailability
Source: Front Microbiol. 2017 Aug 2;8:1424. doi: 10.3389/fmicb.2017.01424 (PMC5539119; doi:10.3389/fmicb.2017.01424)
Supplement: Supplementary file 5 [file DataSheet5.PDF]

Protein	Function	*A. niger* protein identifier		Expression (avNtCov ± sd)	3log2FC (read count) vs NW305 -Fe	
		[1]CBS 513.88	[2]ATCC 1015	NW305 -Fe	NW305 ++Fe	NW186 -Fe
**Iron siderophore biosynthesis (SB)**						
SidA/Sid1	Ornithine monooxygenase	An05g00220	1118819	(429.39 ± 36.37)	-6.60 (4.83 ± 0.51)	-0.40 (292.64 ± 16.28)
SidC/Sid2	Ferricrocin/ferrichrome NRPS	An06g01300	1189171	(16.25 ± 3.83)	-1.91 (5.48 ± 0.42)	0.13 (15.81 ± 1.48)
SidD	Fusarinine C/coprogen NRPS	An03g03520	1186498	(42.80 ± 11.49)	-4.09 (2.72 ± 0.66)	-0.41 (28.68 ± 1.35)
SidF	Transacylase	An03g03540	1109689	(128.15 ± 8.48)	-7.90 (0.54 ± 0.09)	-0.18 (101.98 ± 19.15)
SidG	Transacetylase	-	-	-	-	-
SidH	Mevalonyl-CoA hydratase	An03g03550	1186501	(102.36 ± 6.69)	-8.26 (0.30 ± 0.05)	0.06 (95.71 ± 3.03)
SidI	Mevalonyl-CoA ligase	An06g01320	1160936	(129.09 ± 16.06)	-7.85 (0.50 ± 0.14)	-0.21 (100.37 ± 13.34)
SidL	Transacetylase	An01g03300	1116897	(42.57 ± 7.25)	-0.01 (45.59 ± 3.12)	0.69 (61.85 ± 3.37)
NpgA/PptA	Phosphopantetheinyl transferase	An12g03950	1143106	(14.81 ± 0.43)	-0.06 (15.65 ± 2.45)	0.11 (14.36 ± 0.98)
EstB	Triacetylfusarinine C (TAFC) esterase	-	-	-	-	-
Lipase/Esterase	Putative SB lipase/esterase	An03g03530	1092812	(306.12 ± 4.01)	-6.85 (2.82 ± 0.11)	0.22 (318.38 ± 19.30)
**Iron siderophore transport**						
MirA	Enterobactin transporter	-	-	-	-	-
MirB	Triacetylfusarinine C/coprogen B transporter	An03g03560	1146101	(343.26 ± 69.92)	-9.01 (0.71 ± 0.05)	-0.25 (259.78 ± 1.77)
MirC	Putative iron siderophore transporter	An02g14190	1165578	(110.33 ± 11.86)	-1.20 (53.59 ± 0.79)	0.19 (112.62 ± 0.55)
MirD	Putative iron siderophore transporter	An07g06240	1105147	(202.52 ± 38.35)	-6.32 (2.88 ± 0.05)	1.15 (407.77 ± 8.54)
SitT	ABC transporter	An03g03620	1109705	(93.13 ± 18.69)	-6.38 (1.20 ± 0.18)	-0.35 (65.19 ± 4.18)
**Reductive iron assimilation (RIA)**						
FtrA	Iron permease	An16g01130	1217505	(0.53 ± 0.01)	-	-
		An01g08950	1142522	(253.60 ± 13.40)	-5.68 (5.10 ± 0.01)	-0.30 (184.01 ± 18.45)
		An15g05510	1134159	(2.91 ± 0.02)	-2.38 (0.59 ± 0.11)	2.10 (11.38 ± 1.57)
FreB	Ferric reductase	An11g00220	1126967	(23.56 ± 1.47)	-2.13 (5.98 ± 0.09)	0.18 (23.87 ± 0.67)
		An16g01150	1091043	(0.13 ± 0.03)	-	-
		An13g02180	1081543	(0.02 ± 0.01)	-	-
		An10g00310	1123184	(12.71 ± 1.64)	0.83 (24.53 ± 1.39)	-0.96 (5.76 ± 0.32)
	Putative low affinity iron permease		1145780	(55.04 ± 5.10)	-0.61 (39.93 ± 8.16)	-1.69 (15.35 ± 0.44)
	Putative metalloreductase/ferric-(chelate) reductase activity		1115362	(3.65 ± 0.05)	6.82 (466.27 ± 18.76)	4.41 (69.97 ± 24.19)
			1156801	(14.37 ± 1.96)	0.23 (18.53 ± 1.20)	-0.35 (10.11 ± 0.37)

Protein	Function	*A. niger* protein identifier		Expression (avNtCov ± sd)	3log2FC (read count) vs NW305 -Fe	
		[1]CBS 513.88	[2]ATCC 1015	NW305 -Fe	NW305 ++Fe	NW186 -Fe
	Putative metalloreductase/ferric-(chelate) reductase activity - cont.		1119237	(18.67 ± 1.46)	3.74 (279.22 ± 11.48)	2.46 (91.05 ± 15.23)
			1157566	(1.33 ± 0.12)	-4.49 (0.06 ± 0.03)	1.09 (2.65 ± 0.15)
			1164388	(1.63 ± 0.24)	5.12 (55.86 ± 3.18)	3.87 (19.05 ± 1.79)
			1185315	(38.27 ± 0.78)	2.11 (183.00 ± 13.76)	1.63 (106.32 ± 3.12)
			1158250	(2.94 ± 0.10)	1.20 (7.39 ± 0.06)	-0.47 (1.86 ± 0.22)
			1123184	(12.71 ± 1.64)	0.83 (24.53 ± 1.39)	-0.96 (5.76 ± 0.32)
			1220516	(7.00 ± 0.10)	0.48 (10.63 ± 0.13)	-0.56 (4.28 ± 0.73)
	NADPH oxidase		1155382	(11.88 ± 0.76)	0.64 (20.46 ± 2.22)	0.48 (14.75 ± 2.99)
FetC	Ferroxidase	An01g08960	1142524	(217.48 ± 18.30)	-8.43 (0.64 ± 0.11)	-0.18 (172.51 ± 8.58)
		An15g05520	1134160	(0.21 ± 0.04)	-	2.32 (1.04 ± 0.02)
		An14g05370	1156112	(1.86 ± 0.20)	0.50 (2.74 ± 0.25)	-0.67 (0.97 ± 0.18)
**Regulatory proteins/transcription factors (TF)**						
AcuM	Zn_2Cys_6 TF; Repression of iron uptake including SB and RIA	An02g04370	1184770	(20.75 ± 2.02)	-0.37 (17.47 ± 0.73)	-0.14 (17.09 ± 2.53)
HapX	bZip TF; Repression of iron consumption, activation of iron uptake	An09g06280	1001547	(133.73 ± 17.79)	-1.74 (44.57 ± 3.11)	-0.74 (72.35 ± 2.62)
MpkA	MAP kinase A; Repression of SB	An01g09520	205706	(86.97 ± 5.29)	-0.43 (70.76 ± 5.92)	-0.33 (61.45 ± 3.71)
PacC	$(Cys_2His_2)_3$ TF; Activation of TAFC biosynthesis in alkaline pH	An02g07890	1184997	(24.63 ± 0.48)	0.72 (45.21 ± 1.71)	1.53 (64.19 ± 1.30)
SrbA	bHLH-LZ TF; Activation of iron uptake including SB and RIA	An03g05170	1177110	(32.03 ± 6.94)	-0.58 (23.42 ± 2.02)	0.80 (49.60 ± 6.36)
	bHLH DNA binding domain		1181156	(1970.48 ± 217.65)	-1.15 (986.22 ± 199.26)	1.32 (4409.21 ± 597.09)
SreA	GATA TF; Repression of iron uptake including SB and RIA	An01g02370	1181829	(33.65 ± 1.19)	0.82 (66.02 ± 3.24)	1.25 (71.48 ± 2.19)

[1]As identified by Franken *et al.* (see reference in main text).

[2]Best bi-directional Blast hit CBS 513.88 (see above) with ATCC 1015, unless no CBS 513.88 homologue was identified.

[3]read counts for FC calculation, expression (avNtCov ± sd) given in brackets
