# Supplementary material for: Aspergillus niger Secretes Citrate to Increase Iron Bioavailability
Source: Front Microbiol. 2017 Aug 2;8:1424. doi: 10.3389/fmicb.2017.01424 (PMC5539119; doi:10.3389/fmicb.2017.01424)
Supplement: Supplementary file 6 [file DataSheet6.DOCX]

Supplementary Material

*Aspergillus niger* secretes citrate to increase iron bioavailability

Dorett I Odoni, Merlijn P van Gaal, Tom Schonewille, Juan A Tamayo-Ramos, Vitor A P Martins dos Santos, Maria Suarez-Diez and Peter J Schaap^*^

*** Correspondence:** Corresponding Author: peter.schaap@wur.nl

**Table S1.** Total siderophore concentration [mg/L] of *A. niger* NW305 and NW186 grown with varying iron concentrations in the medium (Fe source: Fe(II)SO_4_, N source: NaNO_3_).

| Strain (major organic acid(s) produced) | Fe added | t = 0 | t = 24 | t = 48 | t = 72 | t = 96 |
| --- | --- | --- | --- | --- | --- | --- |
| NW305 (oxalate, citrate) | - | 0.75 ± 0.60 | 11.74 ± 0.13 | 23.53 ± 0.47 | 73.53 ± 18.63 | 50.46 ± 2.48 |
|  | + | - | 12.367 ± 0.92 | 39.43 ± 3.70 | 72.55 ± 11.77 | 63.73 ± 0.98 |
|  | ++ | 0.15 ± 0.15 | 4.91 ± 2.72 | 16.43 ± 0.84 | 25.21 ± 1.75 | 23.03 ± 1.33 |
| NW186 (citrate) | - | 1.62 ± 0.57 | 7.59 ± 0.45 | 44.57 ± 2.83 | 44.17 ± 0.81 | 45.09 ± 4.73 |
|  | + | 0.30 ± 0.30 | 6.68 ± 1.36 | 15.96 ± 1.81 | 23.64 ± 1.71 | 22.54 ± 2.01 |
|  | ++ | 0.675 ± 0.675 | - | 4.61 ± 1.85 | 6.10 ± 0.33 | 6.443 ± 0.277 |
